# Dynamic leaf energy balance: deriving stomatal conductance from thermal imaging in a dynamic environment

**DOI:** 10.1093/jxb/erz068

**Published:** 2019-02-22

**Authors:** Silvere Vialet-Chabrand, Tracy Lawson

**Affiliations:** School of Biological Sciences, University of Essex, Colchester, UK

**Keywords:** Boundary layer, energy balance, patchy stomata, phenotyping, stomatal conductance, thermal imaging, transpiration

## Abstract

In spite of the significant progress made in recent years, the use of thermography to derive biologically relevant traits remains a challenge under fluctuating conditions. The aim of this study was to rethink the current method to process thermograms and derive temporal responses of stomatal conductance (*g*_sw_) using dynamic energy balance equations. Time-series thermograms provided the basis for a spatial and temporal characterization of *g*_sw_ responses in wheat (*Triticum aestivum*). A leaf replica with a known conductance was used to validate the approach and to test the ability of our model to be used with any material and under any environmental conditions. The results highlighted the importance of the co-ordinated stomatal responses that run parallel to the leaf blade despite their patchy distribution. The diversity and asymmetry of the temporal response of *g*_sw_ observed after a step increase and step decrease in light intensity can be interpreted as a strategy to maximize photosynthesis per unit of water loss and avoid heat stress in response to light flecks in a natural environment. This study removes a major bottleneck for plant phenotyping platforms and will pave the way to further developments in our understanding of stomatal behaviour.

## Teaser

Spatiotemporal co-ordination of the stomatal response to light intensity over the lamina of different wheat leaves was assessed using a novel dynamic energy balance model.

## Introduction

After a period of increased crop production over the past 50 years (Wik *et al.*, 2008; Pingali, 2012; Tillman *et al.*, 2015), increases in yield have fallen to ~1% per annum (Fischer and Edmeades, 2010; Ramankutty *et al.*, 2018). To meet the increasing food demand, crop yield needs to increase at a rate of 2.4% per annum over the next few decades (Tilman *et al.*, 2011; Ray *et al.*, 2012, 2013; Long *et al.*, 2015). The expanding gap between crop production and demand will drive more acute food insecurity across the globe that will only intensify with global population growth (Long *et al.*, 2015; FAO *et al.*, 2018). Along with new agronomic practices, the development of innovative breeding techniques and novel trait discoveries are required to improve yield. Many of the current commercial wheat cultivars have been bred with yield as the main target trait, an approach that is now reaching its limit in term of potential improvement (Fischer and Rebetzke, 2018). Breeding for yield has resulted in a loss of diversity in wheat (Haudry *et al.*, 2007) and in the indirect selection for traits that contribute to higher yields (Reynolds *et al.*, 1999; Richards, 2000; Fischer and Rebetzke, 2018). The natural genetic diversity between cultivars or landraces provides the opportunity to discover desirable traits (e.g. resistance to pest or stress; high water use efficiency) that, when crossed with high-yielding varieties, could produce progenies with improved performance and yield under a given environment. However, identifying individuals with the desired eco-physiological and agronomic responses and traits requires the development of appropriate phenotyping tools (Furbank and Tester, 2011; Reynolds *et al.*, 2018).

Plant phenotyping to characterize diversity in physiological traits often requires measuring large numbers of plants ideally under controlled (e.g. glasshouse) and sometimes under non-controlled environmental (e.g. field) conditions to identify those plants with specific allelic combinations that result in the phenotypes with increased agronomic value (Fiorani and Schurr, 2013; Fahlgren *et al.*, 2015). In the last decade, significant advances have been made in genomics resources and tools that have greatly increased our capacity to understand and detect genetic diversity. However, phenotyping approaches have not advanced at the same pace and have been reported in the literature as a major technical bottleneck (Furbank and Tester, 2011; Fiorani and Schurr, 2013; Fahlgren *et al.*, 2015). High-throughput phenotyping platforms have been developed to address some of this shortfall, but can still be limited in the type and rapidity of the measurement that can be undertaken (Li *et al.*, 2014; Rahaman *et al.*, 2015). Further improvements in plant phenotyping methods to match the progress made in genetics are required to identify new traits increasing crop yield in the near future (Furbank and Tester, 2011; McCouch, 2013; Prashar and Jones, 2014).

High-throughput phenotyping of leaf traits often exploits imaging techniques that permit rapid and remote measurements of plants, to assess differences in development, morphological features, pigment concentration, and responses to the environment. Phenotyping platforms typically use a combination of monochromatic, RGB, thermal, and multispectral cameras providing signals that are often an indirect measure of the physiological plant response/traits (e.g. chlorophyll fluorescence as an indicator of the photosynthesis rate). In most cases, interpretation of the raw measurement signal requires extra steps to convert the raw data into biologically significant findings or responses (Fahlgren *et al.*, 2015; Ghanem *et al.*, 2015). For example, leaf temperature measured using thermography requires leaf energy balance equations to derive leaf transpiration (*E*) and stomatal conductance to water vapour (*g*_sw_) (Jones, 2013). Leaf stomatal conductance is often considered an important trait for future yield improvements and is of great interested to physiologists, as stomatal behaviour influences traits such as photosynthetic CO_2_ uptake, water loss, and leaf temperature (Reynolds *et al.*, 1999; Richards, 2000; Rebetzke *et al.*, 2001; Munns *et al.*, 2010; Fischer and Rebetzke, 2018), all of which impact crop performance. Recent studies examining stomatal behaviour have highlighted that slow stomatal responses to changing environmental stimuli can limit photosynthesis rates (*A*) and result in unnecessary water losses, decreasing productivity and plant water use efficiency (Lawson *et al.*, 2010; Lawson and Blatt, 2014; McAusland *et al.*, 2016; Matthews *et al.*, 2018). Reduced stomatal limitation of photosynthesis under a dynamic environment has been shown to impact plant growth and biomass significantly (Pearcy and Way, 2012; Way and Pearcy, 2012;McAusland *et al.*, 2016; Vialet-Chabrand *et al.*, 2017*b*), and therefore could provide a novel breeding target to improve yield (Lawson and Blatt, 2014). However, currently there are limited phenotyping tools capable of exploring variation in stomatal behaviour and kinetic responses (McAusland *et al.*, 2013). Although leaf temperature and thermography have been used successfully in the past to identify mutants with altered stomatal apertures in response to drought, air relative humidity, [CO_2_], or light intensity (Merlot *et al.*, 2002; Wang *et al.*, 2004; Costa *et al.*, 2015; Takahashi *et al.*, 2015), these studies have focused on steady measurements of leaf temperature. Deriving *g*_sw_ from leaf temperature kinetics under a dynamic environment is a challenge that has currently not been addressed, but is crucial to understand time-integrated variations of plant carbon and water budget.

Despite the excellent reports on the practical use of infrared thermometry to estimate *E* and *g*_sw_ (Jones, 1999; Leinonen *et al.*, 2006; Guilioni *et al.*, 2008; Maes *et al.*, 2016; Jones *et al.*, 2018), these are limited to steady-state assumptions that are difficult to support under a dynamic environment (Jones and Leinonen, 2003). Deriving *g*_sw_ from thermography is generally performed by applying a reworked version of the Penman–Monteith equation describing the sum of radiative energy received or lost by the leaf, as well as mass transfer processes to the atmosphere (Monteith and Unsworth, 2012). Simplifications have been proposed in the literature that involve using reference material (dry/wet leaf replica with similar optical properties) to eliminate the need for longwave radiation and/or humidity measures (Leinonen *et al.*, 2006; Guilioni *et al.*, 2008). Although energy balance equations are a mechanistic description of the processes involved in changes of leaf temperature, some energy fluxes (e.g. longwave radiation from the surrounding environment) and properties of the material studied (e.g. absorbance and emissivity) are difficult to evaluate, particularly in the confined space of a phenotyping platform. A significant step toward the use of thermal imaging to derive stomatal behaviour would be to redefine the Penman–Monteith combination equation for use under dynamic environmental conditions and associate it with a statistical approach to estimate the parameters that cannot be assessed without specific laboratory equipment.

To simplify the usage of energy balance equations, the difference in temperature between a transpiring and non-transpiring leaf can be used to calculate the amount of energy lost by transpiration (latent heat). Several methods have been investigated to measure the temperature of a non-transpiring leaf using dry reference materials that replicate the leaf optical properties (green paper or fabrics) or application of grease to the leaf surface to prevent transpiration (Leinonen *et al.*, 2006; Guilioni *et al.*, 2008). Although these methods appear as an interesting option, there is no guarantee that the reference material has the same optical (e.g. absorbance and reflectance) and thermal properties (e.g. specific heat capacity and emissivity) as the intact leaf. This means that under changing environmental conditions, the leaf and reference material temperatures could behave differently, introducing bias and error in estimating *g*_sw_ from thermographs (Jones and Leinonen, 2003; Jones, 2013). Instead of attempting to mimic leaf properties, a more robust approach would be to directly include differences in thermal and optical properties in the energy balance model and predict leaf thermal kinetics from a reference material.

The energy balance model allows an estimation of energy loss by transpiration, which is dependent on the gradient of water vapour from the leaf to the atmosphere and the total conductance to water vapour (*g*_tw_, the ease with which water vapour diffuses through stomatal pores and the boundary layer). As *g*_tw_ is the combination of two conductances in series, *g*_sw_ and the boundary layer conductance (*g*_bw_), it is possible to derive *g*_sw_ from *g*_tw_ if *g*_bw_ is known. A common method used to estimate *g*_bw_ consists of applying a cycle of heating/cooling to a leaf replica (similar shape) resulting in an exponential increase or decrease in temperature from which *g*_bw_ can be derived using the value of the slope (see details in Jones, 2013). Although this method provides an estimate of the boundary layer, it can only provide a value after several minutes of response when the environmental conditions are relatively stable (Leuning *et al.*, 1989; Leuning and Foster, 1990; Brenner and Jarvis, 1995; Stokes *et al.*, 2006; Katsoulas *et al.*, 2007). Here, we propose the use of a passive method relying only on the difference in energy balance observed under a dynamic environment of references with different thermal properties to estimate the boundary layer conductance.

Rearranging and solving the Penman–Monteith combination equation for *g*_sw_ supposes that the energy budget is closed (i.e. that all the fluxes are taken into consideration in the thermal budget), and integrates the error on each thermal flux in the calculation of *g*_sw_. An alternative approach is to predict leaf temperature using an energy balance model with an in-built dynamic model of *g*_sw_, which, once fitted on the observed temperature, will provide the temporal kinetics of *g*_sw_, as well as key parameter values related to the leaf thermal response. Using Bayesian inference, the probability of each parameter value included in the energy balance model and the prediction errors can be characterized for different individuals, enabling a more precise quantification of the trait diversity associated with the parameters.

The aim of this study was to provide a method to process thermograms describing leaf temperature kinetics under fluctuating conditions and derive temporal responses of stomatal conductance (*g*_sw_), using a new interpretation of energy balance equations. The results deliver important information on the diversity of the leaf responses to changes in light intensities, providing a breakthrough in data processing for phenotyping. Model performance and its application for plant phenotyping are presented, as well as a description of the biological importance of the response traits estimated.

## Materials and methods

The Penman–Monteith equation (Penman, 1948; Monteith, 1965) combines the surface energy balance with mass transfer (the transport of water vapour from the leaf to the atmosphere) and was originally developed to compute transpiration from cropped surfaces using commonly measured weather data (solar radiation, air temperature, vapour content, and wind speed). Under the assumption of steady-state environmental conditions, the Penman–Monteith combination equation can be solved for leaf stomatal conductance (Monteith and Unsworth, 2012; Jones, 2013) and is therefore widely used in association with thermography to study plant response. Here, we further develop this equation by including temporal effects that affect thermal exchange between the leaf and its environment using differential equations. Although such equations are more complex to use and solve, they are essential to understand the dynamics of leaf temperature and water loss under a changing environment.

### Energy balance model under steady-state environmental conditions

**Fig. 1.**
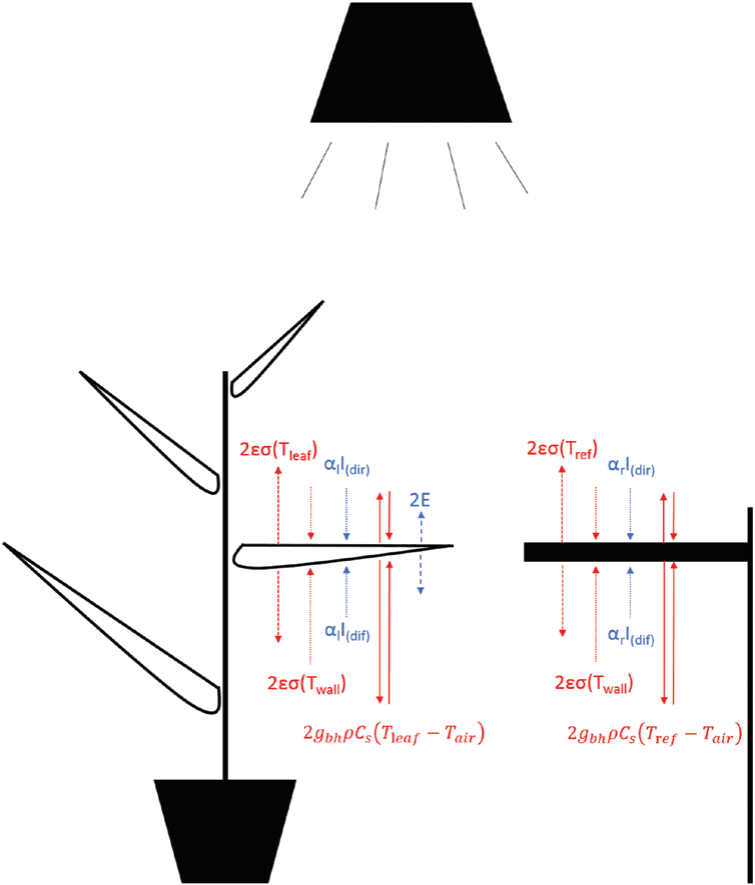
Schematic of a leaf and an aluminium reference energy budget in the enclosed area of a phenotyping platform. The aluminium reference was painted black and had an emissivity (ε) of 0.96 to act as a reference for leaf energy balance assessment. Each arrow represents an energy flux, with the arrow direction representing either an input or output of energy. Symbol definition: σ, Stefan–Boltzmann constant; *T*_leaf_, leaf temperature; *T*_wall_, wall temperature; *T*_ref_, aluminium reference temperature; *T*_air_, air temperature; *I*_(dir)_, direct irradiance; *I*_(dif)_, diffuse irradiance; α_l_, leaf absorbance to shortwave irradiance; α_r_, reference absorbance to shortwave irradiance; *g*_bh_, boundary layer conductance to heat transfer; ρ, air density; *C*_s_, specific heat capacity of humid air; *E*, leaf transpiration. Factor 2 in the equations represent the input or output energy for the two sides of the leaf/reference.

Under steady-state environmental conditions, leaf temperature is at equilibrium and therefore the sum of the radiative energy received and lost by the leaf is null (Fig. 1; see Table 1 for symbol description and units). Any imbalance in the energy fluxes due to change in environmental conditions impacts the energy stored by the leaf, and results in variation in leaf temperature. Neglecting any metabolic storage (heat stored as chemical bond energy, e.g. photosynthesis), the energy balance equation reduces to:

**Table 1.** Energy balance parameters, environmental variables, and physical constants

Symbol	Description	Unit
Gas exchange		
*A*	Net CO_2_ assimilation rate	µmol m^–2^ s^–1^
*g*_sw_	Stomatal conductance to water vapour	mol m^–2^ s^–1^
*g*_bw_	Boundary layer conductance to water vapour	mol m^–2^ s^–1^
*g*_bh_	Boundary layer conductance to heat transfer	m s^–1^
*g*_tw_	Total conductance to water vapour	mol m^–2^ s^–1^
*E*	Transpiration rate	kg m^–2^ s^–1^
*VPD*_l_	Leaf to air vapour pressure deficit	Pa
*g*_1_, *g*_2_, g_*3*_	Steady-state targets for *g*_sw_ for the dark/light/dark periods	mol m^–2^ s^–1^
ϕ_i_, ϕ_d_	Time lag for an increase (_i_) or a decrease (_d_) in *g*_sw_	s
τ_i_, τ_d_	Time constant for an increase (_i_) or a decrease (_d_) in *g*_sw_	s^–1^
*S*_l_	Slope of the linear variation of *g*_sw_ during the light period	µmol m^–2^ s^–2^
Energy balance		
*T*_leaf_	Leaf temperature	°C/°K
*R*_n_	Net radiation	W m^–2^
*C*	Sensible heat transfer	W m^–2^
λ	latent heat of evaporation of water	J kg^–1^
*S*	Net physical storage	W m^–2^
α_l_, α_b_, α_w_	Short-wave absorbance of the leaf (_l_), black reference (_b_), and white reference (_w_)	
ε_l_, ε_b_, ε_w_	Emissivity of the leaf (_l_), black reference (_b_), and white reference (_w_)	
*k*	Amount of energy per unit area required to change the temperature of the material by 1 °K	J m^–2^ K^–1^
ρ, ρ*	Density of air and leaf tissue (*)	kg m^–3^
*C*_s_	Specific heat capacity of humid air	J kg^–1^ K^–1^
*C*_p_*	Leaf specific heat capacity	J kg^–1^ K^–1^
l*	Leaf thickness	m
Environment		
*I*_s_	Incident shortwave radiations	W m^–2^
PPFD	Photosynthetic photon flux density	µmol m^–2^ s^–1^
RH	Air relative humidity	
*T*_air_	Air temperature	°C/°K
*P*_atm_	Atmospheric pressure	Pa
*e*_s_, *e*_a_	Leaf internal (_s_) and air (_a_) vapour pressure	Pa
Constant		
θ	Stefan–Boltzmann constant	W m^–2^ K^–4^
*R*	Gas constant	m^3^ Pa K^−1^ mol^−1^

$$\begin{array}{l} R_{n}-C-\lambda E=S \end{array}$$ (1)

where *R*_n_ is the net radiation (W m^–2^), *C* is the sensible heat transfer (W m^–2^), λ is the latent heat of evaporation of water (J kg^–1^), *E* is the evaporative flux (kg m^–2^ s^–1^), and *S* is the net physical storage (W m^–2^, causing a change in leaf temperature and equal to 0 under steady-state conditions). After a change in environmental conditions, a delay is required to reach a new equilibrium depending on the material utilized, and therefore Equation 1 may over- or underestimate leaf temperature during the transition from one equilibrium to another.

### Energy balance model under dynamic environmental conditions

Under dynamic environmental conditions, temperature kinetics of an object depend on the thermal and optical properties of the object, which describes how efficiently the energy is captured, exchanged, and stored. A differential equation describing the time dependency of temperature kinetics is available from the literature (Monteith and Unsworth, 2012; Jones, 2013):

$$\frac{\text{d}T}{\text{d}t}=\frac{R_{\text{n}}-C-\text{λ}E}{{\text{ρ}}^{*}C_{\text{p}}^{*}l^{*}}$$ (2)

where ρ* and C*_p_ are the density (kg m^–3^) and specific heat capacity (  J kg^–1^ K^–1^), respectively, of leaf tissue and *l* * is the leaf thickness (m). Steady-state energy balance equations are generally simplified by using the temperature of a reference material mimicking a leaf without transpiration, as described by Jones and colleagues (Leinonen *et al.*, 2006; Guilioni *et al.*, 2008; Jones *et al.*, 2018). The difference in energy balance between the leaf and the reference material (placed in the same orientation as the leaf) allows a simplification of the energy fluxes (see below) to remove most of the effects due to the surrounding environment (e.g. longwave radiation). Including the thermal properties of the material (ρ*, C*_p_, *l* *) in the energy balance model can improve the accuracy of the model but also results in different denominators complicating the equation. Solving the differential equation that describes the difference in energy balance between the leaf and a reference material using Equation 2 can enable the use of any material with known properties as a reference. An advantage of this proposed approach is that instead of trying to mimic the properties of the leaf, the differences in thermal and optical properties between the two objects are directly included in the equations. It is possible to predict the temperature (*T*_1_, °K) of any material (e.g. leaf) using the temperature kinetics of a reference (*T*_2_, °K; e.g. black painted aluminium sheet) with knowledge of the optical and thermal properties of the material. Under a given environment, the temperature kinetics of an object are predicted using the difference in energy balance between the object and the reference, which is described by the following equations:

$$\begin{array}{l} \begin{array}{l} \frac{\text{d}T_{1}}{\text{d}t}=\frac{R_{\text{n}1}-C_{1}-\text{λ}E_{1}}{\text{ρ}*{\text{​}}_{1}C^{*}{}_{\text{p}1}l^{*}{}_{1}}\text{ with ρ}{*}_{1}C^{*}{}_{\text{p}1}l^{*}{}_{1}=k_{1} \end{array} \end{array}$$ (3)

$$\begin{array}{l} \begin{array}{l} \frac{\text{d}T_{2}}{\text{d}t}=\frac{R_{\text{n}2}-C_{2}-\text{λ}E_{2}}{\text{ρ}{*}_{2}C^{*}{}_{\text{p}2}l^{*}{}_{2}}\text{ with ρ}{*}_{2}C^{*}{}_{\text{p}2}l^{*}{}_{2}=k_{2} \end{array} \end{array}$$ (4)

where ρ*_1_ and ρ*_2_ are the density (kg m^–3^), C*_p1_ and C*_p2_ are the specific heat capacity (J kg^–1^), and l*_p1_ and l*_p2_ are the thickness (m) of the objects measured. Therefore, *k*_1_ and *k*_2_ represent the amount of energy per unit area required to change the temperature of the material by 1 °K (J m^–2^ K^–1^). It is important to note that a factor of 2 can be applied to the latent heat term (λ*E*_1/2_) if the object is transpiring from both surfaces.

Rearranging the equation by moving the denominator to the left-hand side results in:

$$\begin{array}{c} k_{1}\frac{\text{d}T_{1}}{\text{d}t}-k_{2}\frac{\text{d}T_{2}}{\text{d}t}=R_{\text{n}1}-C_{1}-\text{ ​​}\lambda \text{​​ }E_{1}-\left(R_{\text{n}2}-C_{2}-\text{ ​​}\lambda \text{​​ }E_{2}\right) \end{array}$$ (5)

This configuration allows further simplification by regrouping similar energy fluxes:

$$\begin{array}{c} k_{1}\frac{\text{d}T_{1}}{\text{d}t}-k_{2}\frac{\text{d}T_{2}}{\text{d}t}=R_{\text{n}1}-R_{\text{n}2}+C_{2}-C_{1}+\text{ ​​}\lambda \text{​​ }E_{2}-\text{ ​​}\lambda \text{​​ }E_{1} \end{array}$$ (6)

If *E*_2_ is for a non-transpiring reference material (e.g. an aluminium replica) and therefore has a value of 0, the equation would be:

$$\begin{array}{c} k_{1}\frac{\text{d}T_{1}}{\text{d}t}-k_{2}\frac{\text{d}T_{2}}{\text{d}t}=R_{\text{n}1}-R_{\text{n}2}+C_{2}-C_{1}-\text{​​}\lambda \text{​​ }E_{1} \end{array}$$ (7)

The net radiation (*R*_n_) for a horizontal two-sided object is defined by its longwave and shortwave radiation exchanges with its environment (Fig. 1):

$$\begin{array}{l} \begin{array}{l} R_{nx}=\;\alpha_{x}I_{s}-2\theta \varepsilon_{x}{T_{x}}^{4}+L_{d} \end{array} \end{array}$$ (8)

where subscript x is either 1 or 2, α_x_ is the absorbance to shortwave radiations (incident and diffuse; *I*_s_, W m^–2^), θ the Stefan–Boltzmann constant (W m^–2^ K^–4^), ε_x_ the emissivity, *T*_x_ the temperature of the object studied (°K), and *L*_d_ the longwave radiation (W m^–2^) received from the surrounding environment (e.g. soil, wall). The factor of 2 in 2θε_x_*T*_x_^4^ correspond to the longwave radiation energy lost by each side of the object. Using a reference material placed under similar conditions to the object measured removes the need to quantify *L*_d_ as it disappears from the equation when the difference in energy flux is calculated (see below). This assumption is valid when the reference material and the leaf are surrounded by a similar thermal environment, with an increasing risk of error when the distance between the two objects increases (e.g. large imaging area).

The sensible heat is the energy lost by conduction and convection and is defined as:

$$C_{\text{x}}=\;2g_{\text{bhx}}\text{ρ}C_{\text{s}}(T_{x}-T_{a})$$ (9)

where *g*_bhx_ is the one-sided boundary layer conductance to heat transfer (m s^–1^), ρ the air density (kg m^–3^), *C*_s_ the specific heat capacity of humid air (J kg^–1^ K^–1^), and *T*_a_ is the air temperature (°K). The factor of 2 in the equation correspond to the energy lost by each side of the object. Air properties such as ρ and *C*_s_ are described in Supplementary Table S1 at *JXB* online.

Substituting Equations 8 and 9 in Equation 7 gives:

$$k_{1}\frac{\text{d}T_{1}}{\text{d}t}-k_{2}\frac{\text{d}T_{2}}{\text{d}t}={\text{α}}_{1}I_{\text{s}}-2{\text{θε}}_{1}T_{1}{}^{4}-{\text{α}}_{2}I_{\text{s}}-2{\text{θε}}_{2}T_{2}{}^{4}+2g_{\text{bh}2}\text{ρ}C_{\text{s}}\left(T_{2}-T_{\text{a}}\right)-2g_{\text{bh}}{}_{1}\text{ρ}C_{\text{s}}\left(T_{1}-T_{\text{a}}\right)-\text{λ}E_{1}$$ (10)

Details required to calculate the different terms are provided in Supplementary Algorithm S1.

Temperature kinetics can be predicted using the following generic equation:

$$\begin{array}{l} \begin{array}{l} \frac{dT_{1}}{dt}=\frac{\begin{array}{c} k_{2}\frac{dT_{2}}{dt}-I_{s}\left(\alpha_{2}-\alpha_{1}\right)+2\theta \left(\varepsilon_{2}{T_{2}}^{4}-\varepsilon_{1}{T_{1}}^{4}\right)\\ +\;2\rho C_{s}\left[\;\;g_{bh2}\left(T_{2}-T_{air}\right)-g_{bh1}\left(T_{1}-T_{air}\right)\right]-\lambda E_{1} \end{array}}{k_{1}} \end{array} \end{array}$$ (11)

where: $k_{2}\frac{dT_{2}}{dt}$ is the change in energy of the reference material per unit of time; *I*_s_(α_2_–α_1_) the difference in absorbed shortwave radiations; 2θ(ε_2_*T*_2_^4^–ε_1_*T*_1_^4^) is the difference in energy lost by longwave radiations; 2ρ*C*_s_[*g*_bh2_ (*T*_2_–*T*_air_)–*g*_bh1_ (*T*_1_–*T*_air_)] is the difference in energy exchange by conduction and convection with the atmosphere; and λ*E*_1_ is the energy lost by transpiration.

Such an equation can be used to calculate *T*_1_ at any given time using an ordinary differential equation (ODE) solver. The solver required Equation 11 to be calculable at any time *t* supposing that the derivative of the reference temperature kinetics (d*T*_2_/d*t*) and the discrete estimates of the environmental variables [*I*_s_, relative humidity (RH), *T*_air_, *P*_atm_] can be interpolated as a function of time. Using a smooth spline on recorded values of *T*_2_ and each environmental variable removes high-frequency noise (making the ODE solver more efficient) and provides functions that for any time *t* return a value and its derivative (Supplementary Fig. S1). Using an ODE solver requires an initial value for the predicted temperature that was included as an estimated parameter in the Bayesian inference described below. Equation 11 is a ‘stiff’ equation requiring a solver with variable step size (e.g. ‘cvode’ at https://computation.llnl.gov/projects/sundials), as standard methods such as Runge-kutta were orders of magnitude slower to solve this equation.

### Leaf transpiration model

In response to changes in leaf energy balance, leaf transpiration varies, regulating leaf temperature to, for example, maintain an optimal range for photosynthesis. Leaf transpiration (*E*) can be calculated using Equation 12:

$$\begin{array}{l} \begin{array}{l} E=\frac{0.622\rho }{P_{atm}}\frac{RT_{leaf}}{P_{atm}}g_{tw}\left(e_{s}-e_{a}\right) \end{array} \end{array}$$ (12)

with $\frac{0.622\rho }{P_{atm}}$ a conversion factor from Pa to kg m^–3^ and $\frac{RT_{leaf}}{P_{atm}}$ a conversion factor from mol m^–2^ s^–1^ to m s^–1^, and where *P*_atm_ is the atmospheric pressure (Pa), *T*_leaf_ the leaf temperature (°K), *g*_tw_ the total conductance to water vapour (mol m^–2^ s^–1^), *e*_s_ the leaf internal vapour pressure, and *e*_a_ the air vapour pressure (Pa).

Total conductance to water vapour (*g*_tw_, mol m^–2^ s^–1^) is the sum in series of the boundary layer conductance to water vapour (*g*_bw_, mol m^–2^ s^–1^) and stomatal conductance to water vapour (*g*_sw_, mol m^–2^ s^–1^):

$$\begin{array}{c} g_{\text{tw}}=\frac{1}{\frac{1}{g_{\text{bw}}}+\frac{1}{g_{\text{sw}}}} \end{array}$$ (13)

where *g*_bw_ depends on leaf morphology and wind speed, and *g*_sw_ depends mainly on the number of stomata and change in aperture in response to the surrounding environment.

Substituting Equations 12 and 13 in Equation 11 allows us theoretically to solve Equation 11 for *g*_sw_, assuming that the different derivatives included in Equations 11 can be extrapolated using splines adjusted on the temperature kinetics. Although estimating the derivative at different time points using a spline is an approach that could be considered, it should be done with caution as it can give unstable *g*_sw_ values due to the sensitivity to measurement noise of the method. Indeed, errors in estimating the derivatives of temperature kinetics from noisy data will propagate in the estimation of *g*_sw_. In the case of the temperature kinetics of the reference material, this error is minimized, as the pattern of variation (exponential increase) is simple to approximate, which is not always the case for the leaf temperature kinetics. Therefore, the approach chosen here was to directly include a model to predict *g*_sw_ in the energy balance equations to calculate the derivative and solve it using an ODE solver, leaving the errors propagated to the predicted temperatures. The prediction errors of the model can thus be taken into consideration when fitting to the observed data.

### Stomatal conductance model

The temporal response of stomatal conductance to water vapour (*g*_sw_, mol m^–2^ s^–1^) can be predicted using a previously described dynamic model (Vialet-Chabrand *et al.*, 2017*b*). In the case of a continuous variation in light intensity, differential equations are required to model *g*_sw_ (Vialet-Chabrand *et al.*, 2013, 2016). However, for a step change in light intensity, such an equation can be solved analytically, resulting in a sigmoidal equation, which is simpler and faster to calculate as a function of time (*t*, s):

$$\begin{array}{c} g_{\text{sw}}=\left(G+s_{\text{l}}\cdot t-g_{0}\right)\frac{e^{-{\text{e}}^{\frac{\phi -\text{t}}{\text{ ​​}\tau \text{​​ }}+1}}-e^{-{\text{e}}^{\frac{\phi }{\text{ ​​}\tau \text{​​ }}+1}}}{1-e^{-{\text{e}}^{\frac{\phi }{\text{ ​​}\tau \text{​​ }}+1}}}+g_{0} \end{array}$$ (14)

Where *g*_0_ is the initial value of *g*_sw_ at *t*_0_=0, *G* is the steady-state target of *g*_sw_, τ the time constant of the response (s), ϕ the initial time lag (s), and *s*_l_ the slope of the slow decrease/increase in *g*_sw_ (mol m^–2^ s^–2^). The term $\left(e^{-e^{\frac{\varphi -t}{\tau }+1}}-e^{-e^{\frac{\varphi }{\tau }+1}}\right)/\left(1-e^{-e^{\frac{\varphi }{\tau }+1}}\right)$ has been included to ensure a proper scaling of the sigmoidal equation between *g*_0_ and *G* even when ϕ is positive. Without this correction at *t*=0, the term $e^{-e^{\frac{\varphi }{\tau +1}}}$ would be positive when ϕ was positive, introducing an offset in the estimation of *g*_0_ and changes in the scale of the response. Plants can biologically control the energy lost by leaf transpiration by dynamically adjusting *g*_sw_ or by adjusting the boundary layer conductance by changing the leaf shape (e.g. curling) over a longer period of time.

### Boundary layer conductance

Using two non-transpiring references mimicking leaf shape and having identical thermal properties (same material), but different optical properties (e.g. black and white) enables Equation 9 to be simplified to determine the boundary layer conductance:

$$\begin{array}{l} \begin{array}{l} \frac{dT_{w}}{dt}=\frac{dT_{b}}{dt}-\frac{I_{s}\left(\alpha_{b}-\alpha_{w}\right)+2\theta \left(\varepsilon_{w}{T_{w}}^{4}-\varepsilon_{b}{T_{b}}^{4}\right)+2g_{bh}\rho C_{s}\left(T_{w}-T_{b}\right)}{k} \end{array} \end{array}$$ (15)

where *T*_*w*_ and *T*_*b*_ are the temperature (°K) of the white and the black references, respectively. Knowing the thermal (*k*, ε_w_, ε_b_) and optical (α_w_, α_b_) properties of the references, it is possible to derive *g*_bh_ (m s^–1^) from Equation 15 by adjusting its value to fit the predicted white reference temperature kinetics to the measured data. Depending on the experimental conditions, it is possible to assume that *g*_bh_ is stable over the measurement period and therefore only one value needs to be estimated. For field applications, it is also possible to include a model predicting the boundary layer conductance from wind speed measurements and the dimensions of the references (Jones, 2013). This enables us to take into consideration the effect of wind speed on leaf energy balance under a dynamic field environment.

### Predicting leaf temperature kinetics and stomatal conductance to water vapour

#### Model implementation

Stomatal conductance kinetics were predicted by fitting a model combining Equations 9–13 on leaf temperature kinetics under a dynamic environment. Using environmental variables as inputs [photosynthetic photon flux density (PPFD), µmol m^–2^ s^–1^; RH, %; *T*_air_, °C; *P*_atm_, Pa], the model predicted stomatal conductance kinetics (Equation 12) in response to changes in light intensity, which, combined with estimation of leaf boundary layer conductance (Equation 13), was used to calculate the transpiration rate (Equations 10–11). The latent heat of evaporation was then calculated and included in the leaf energy balance (Equation 9) to predict leaf temperature variations. Equation 9 was applied twice using two different references (black and white) to calculate leaf temperature kinetics, constraining the estimation of *g*_bh_ as in Equation 13. The two different predictions of leaf temperature were compared simultaneously with the observed lead temperature using Bayesian inference (see Supplementary Algorithm S1).

#### Bayesian inference

Bayesian inference was used to tune parameter values to fit model predictions to observed data. Estimating parameter values of such a model is challenging because each parameter has a range of values that can produce equivalent model outputs depending on the precision of observations and the interaction with other parameters. Using Stan, a statistical modelling platform (http://mc-stan.org/), intervals representing 95% of the probable parameter values (credible interval) were estimated for each parameter. The credible interval included the correlation between parameter and the error due to the noisy data. The model outputs were fitted on the observed data by exploring the parameter space using three Markov chain Monte Carlo (MCMC) with 500 iterations (250 iterations without adaption). All chains converged without divergent transition to the same parameter values (Rhat <1.1), with effective sample size values >200 (Carpenter *et al.*, 2017). Further information on the use of Bayesian inference to find parameter values of ordinary differential equations can be found online at http://mc-stan.org/events/stancon2017-notebooks/stancon2017-margossian-gillespie-ode.html. Originally, Stan did not include the possibility to use spline functions in the Bayesian model. The function was included as an external C++ function wrapping the spline function available from the Boost Library into the template system used by Stan.

A possible problem during the model fitting could come from comparing observed leaf temperature kinetics with predicted temperatures using two different references, which could result in bimodal parameter distributions. However, the convergence toward the observed temperature of both sets of predicted temperatures is mainly dependent on the value of the boundary layer conductance (*g*_bh_, Equation 13), which is adjusted during the Bayesian inference, constraining the value of *g*_bh_. Measurements of *g*_bh_ at different times of the day did not differ significantly and a constant *g*_bh_ was assumed for both references during the experiment to predict leaf gas exchange. Moreover, the conditions that generally influence the boundary layer conductance (e.g. air mixing provided by the fan) were relatively constant within our imaging area.

### Model validation using a leaf replica with constant *g*_sw_

**Fig. 2.**
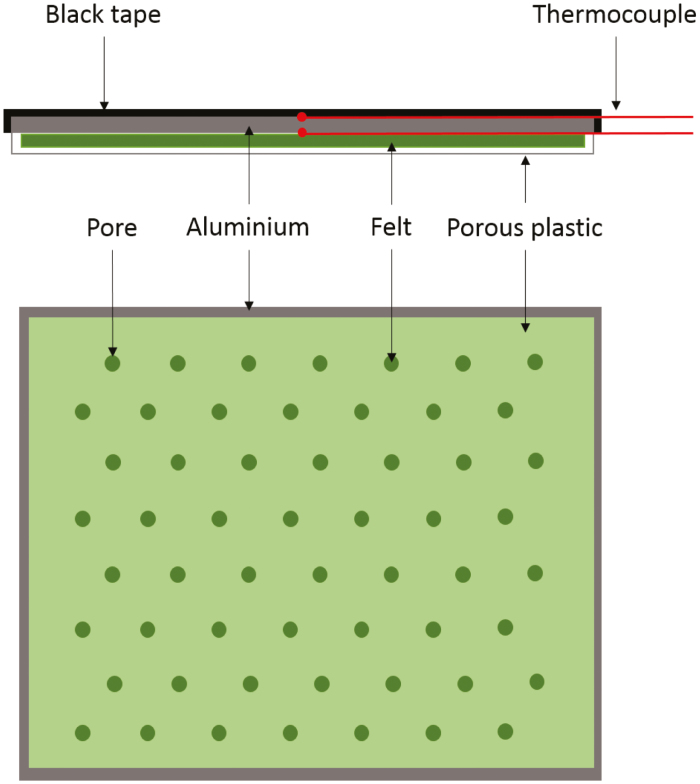
Schematic of the leaf replica used to validate the energy balance equations and experimental set-up. The black tape had an emissivity of 0.97 and an absorbance of 0.96, the aluminium plate provides support with a rapid thermal response, the felt was used as water storage to maintain transpiration through time, and the porous plastic resulted in a constant conductance. The conductance was only dependent on the size of the pores and their distribution across the surface. Two thermocouples were integrated to measure the temperature of the plate on each side.

Under similar conditions to those experienced by plants under step changes in light intensity, accuracy and precision of the model outputs (leaf temperature and conductance) were tested using a reference built to mimic leaf transpiration with a constant conductance to water vapour (Fig. 2). The aim was to compare the conductance predicted by the model, measured using gas exchange and calculated using a physical diffusion equation (Lehmann and Or, 2015). The replica was located at the same position and with the same orientation (horizontal) as the leaves and therefore received the same light intensity (430 μmol m^–2^ s^–1^) as the measured leaves, whilst the aluminium references (black and white) were at a lower location and to one side of the image, and therefore received a slightly lower light intensity of 300 μmol m^–2^ s^–1^. The replica was an aluminium plate covered on one side by black electrical tape with known absorbance and emissivity, and on the other side with a felt fabric enclosed in a plastic microporous sheet. The microporous sheet had pores of a known and standard diameter of 0.5 mm and 40 µm depth, arranged in a grid of 3 mm pitch (density: 160 pores inch^–2^). When the felt was saturated with water, transpiration was dictated by the size and density of the pores, and the variation in environmental conditions (e.g. RH and wind speed). Conductance was calculated using pore dimension and a one end correction (Brown and Escombe, 1900; Cowan, 1978; Weyers and Meidner, 1991). The values from two thermocouples were used to measure the internal temperature of the replica attached on each side and compared with direct measurements from thermal imaging (Supplementary Fig. S2). Temperature measured using thermal imaging resulted in average temperature kinetics from both sides and was used to fit the energy balance model and predict the conductance. The value of conductance determined by fitting the energy balance model on a step increase and decrease in light intensity was validated on an independent data set. In addition, the value was compared with conductance measured using a Li-Cor 6800 (Li-COR Biosciences, Lincoln, NE, USA) and a 9 cm^2^ chamber under steady-state conditions (PPFD, 0 µmol m^–2^ s^–1^; [CO_2_], 400 µmol mol^–1^; *T*_air_, 20 °C; RH, 60%; flow, 500 µmol s^–1^), and calculated from the anatomy of the transpirating surface (density and size of the pores).

### Thermal imaging

Thermal images were recorded using bespoke designed software that continuously records the environmental variables, as well as the temperatures, and performed an emissivity correction for the regions of interest (see below). Black and white references were cut from the same piece of aluminium (width, 2 cm; length, 10 cm; thickness, 0.95 mm) and used as references in each captured picture. The temperature of the black reference was also measured with a surface thermocouple, allowing a comparison of the precision and accuracy of the estimates provided by the thermal camera. A temperature correction was performed using the method described in the manual of the thermal camera in which a crumbled piece of aluminium foil was used to correct each image for the influence of the reflected thermal emission from ambient sources. Images were saved every 3 s and each was an average of 100 raw images on a pixel-by-pixel basis, removing random noise. Leaf isolation was performed by thresholding the leaf area on the captured picture with the highest temperature difference between the air and the black reference (i.e. contrast).

### Set-up validation

The thermal camera used in these experiments was a FLIR A655sc (FLIR system AB, Täby, Sweden) including an uncooled microbolometer detector (resolution, 640×480 pixels; spectral range, 7.5–14.0 µm; noise equivalent temperature difference, <30 mK) and autocalibrates with surrounding temperature. The light was provided by two identical LED light sources (LX601C, Heliospectra AB, Göteborg, Sweden) located on each side of the thermal camera. After a step change in light intensity, air temperature increased and induced artefact in temperature measurements using the thermal camera. To compensate for temperature drifts, a process called non-uniformity calibration was performed every minute, which calculated a new table of correction factors using a miniature black body that moved in front of the detector (Olbrycht and Więcek, 2015). Additionally, temperature from a black aluminium reference was measured using the thermal camera and compared with surface thermocouple measurements to check if the thermal kinetics were accurately captured (Supplementary Fig. S3).

### Plant material

Winter wheat (*Triticum aestivum* L.) plants were grown in 20-well propagators under well-watered conditions in peat-based compost (Levingtons F2S; Everris) for 2 weeks, and were vernalized for 6 weeks in a cold room at 5 °C. Plants were potted in 200 ml pots and moved to a controlled-environment chamber (Photon Systems Instruments, Brno, Czech Republic) with 300 µmol m^–2^ s^–1^ of light intensity (10 h/14 h) provided by white LEDs, temperature of 22/18 °C, and an RH of 50/65% (day/night). The third fully developed leaf of the main tiller was used for measurements.

### Thermal kinetics in response to a step change in light intensity

Plants were dark acclimated for 1 h and then thermal pictures were recorded for 10 min. After this period, plants were subjected to a step change in light intensity from 0 µmol m^–2^ s^–1^ to 430 µmol m^–2^ s^–1^ for 1 h, followed by an opposite step from 430 µmol m^–2^ s^–1^ to 0 µmol m^–2^ s^–1^ also for 1 h. Photon flux was determined with a quantum sensor (SKP 215; Skye Instruments Ltd, Llandrindod Wells, UK) placed near the reference materials, and was converted to energy using a radiation conversion factor measured with a spectroradiometer (model SR9910-PC, Macam Photometrics Ltd, Livingstone, UK). During the experiments, air temperature and RH (Supplementary Figs S3, S5) were recorded simultaneously with the thermal measurements. To ensure even illumination, leaves were attached to a support (length/width, 13/34 cm) that held them flat and horizontal to the light source ensuring no contact with support material. A mixing fan was installed inside the room to provide good air mixing and rapid thermal exchanges, and to prevent creation of local temperature and humidity gradients. The air mixing also increased boundary layer conductance and improved the reactivity of thermal changes, enabling the more efficient capture of temperature variations with the thermal camera.

## Results

### Validation of the energy balance equation and experimental approach

**Fig. 3.**
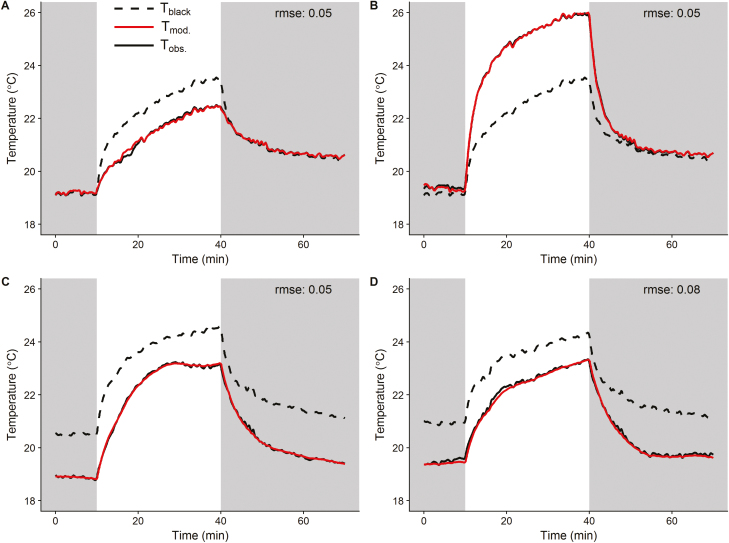
Temperatures of the reference materials and leaf replica observed (solid black line) and modelled (solid red line) in response to a step change in light intensity from dark to light (0–300 µmol m^–2^ s^–1^ at the site of the aluminium references and 0–430 µmol m^–2^ s^–1^ at the site of the leaf replica). Modelled temperatures were derived from the temperature kinetics of a black reference (*T*_black_, dashed black line). Observations described the temperature kinetics of (A) a white reference, (B) a dry leaf replica, and (C) and (D) a wet leaf replica. Parameter values were adjusted using Bayesian inference to minimize the error between observed and modelled data in (A), (B), and (C). Parameter values estimated in (C) were used to predict the temperature kinetics in (D) to test model predictions using an independent data set. The root mean square error (rmse) was calculated as an estimator of the quality of the model outputs.

Various materials (e.g. white reference and leaf replica) were used to validate the experimental approach by evaluating the error between modelled and observed temperature kinetics. Figure 3 shows how close the temperature kinetics predicted using the black reference were to the observed responses from the different materials subjected to a step increase and decrease in irradiance. The thermal kinetics of the white and black references displayed stable temperature during the initial dark period, followed by an exponential increase after the increase in light intensity and an exponential decrease when light was returned to zero (Fig. 3A). The energy balance model accurately described the thermal kinetics of the white reference [root mean square error (rmse) 0.05 °C] and the observed difference between the white and black temperature during the illumination period that was due to the difference in energy absorbed by the two references. The temperature kinetics of the dry leaf replica followed the same pattern of temperature variations and was accurately predicted by the energy balance model using the same black reference (Fig. 3B, rmse 0.05 °C). The differences in temperatures values observed here were due to differences in light intensity received by the two objects located at different positions under light (300 µmol m^–2^ s^–1^ for the black reference, 430 µmol m^–2^ s^–1^ for the dry leaf replica) rather than absorbance as for the references. Transpiration from the wet leaf replica resulted in a large decrease in measured (and predicted) temperature compared with the dry leaf replica; however, a conserved pattern of temperature variations was observed (Fig. 3C). The energy balance model accurately described the thermal kinetics of the wet leaf replica by including energy loss by transpiration (Fig. 3C, rmse 0.05 °C). Parameter values of the energy balance equations were estimated by fitting the predicted temperature kinetics of the wet leaf replica on observed data. Using an independent data set and the same parameter values, the model accurately predicted the wet leaf replica thermal kinetics, validating the model predictions (Fig. 3D, rmse 0.08 °C). The pore conductance of the leaf replica was calculated from the size and distribution of the pores at 0.207 mol m^–2^ s^–1^, and was confirmed by infrared gas analysis at 0.210±0.0008 SD mol m^–2^ s^–1^. Pore conductance derived from thermal kinetics using Bayesian inference had a value of 0.207±0.0008 SD mol m^–2^ s^–1^ (Fig. 3C, D), demonstrating the predictive power and accuracy of the energy balance model.

### Spatiotemporal variability of thermal kinetics in response to a step change in light intensity

#### Description of leaf temperature kinetics

**Fig. 4.**
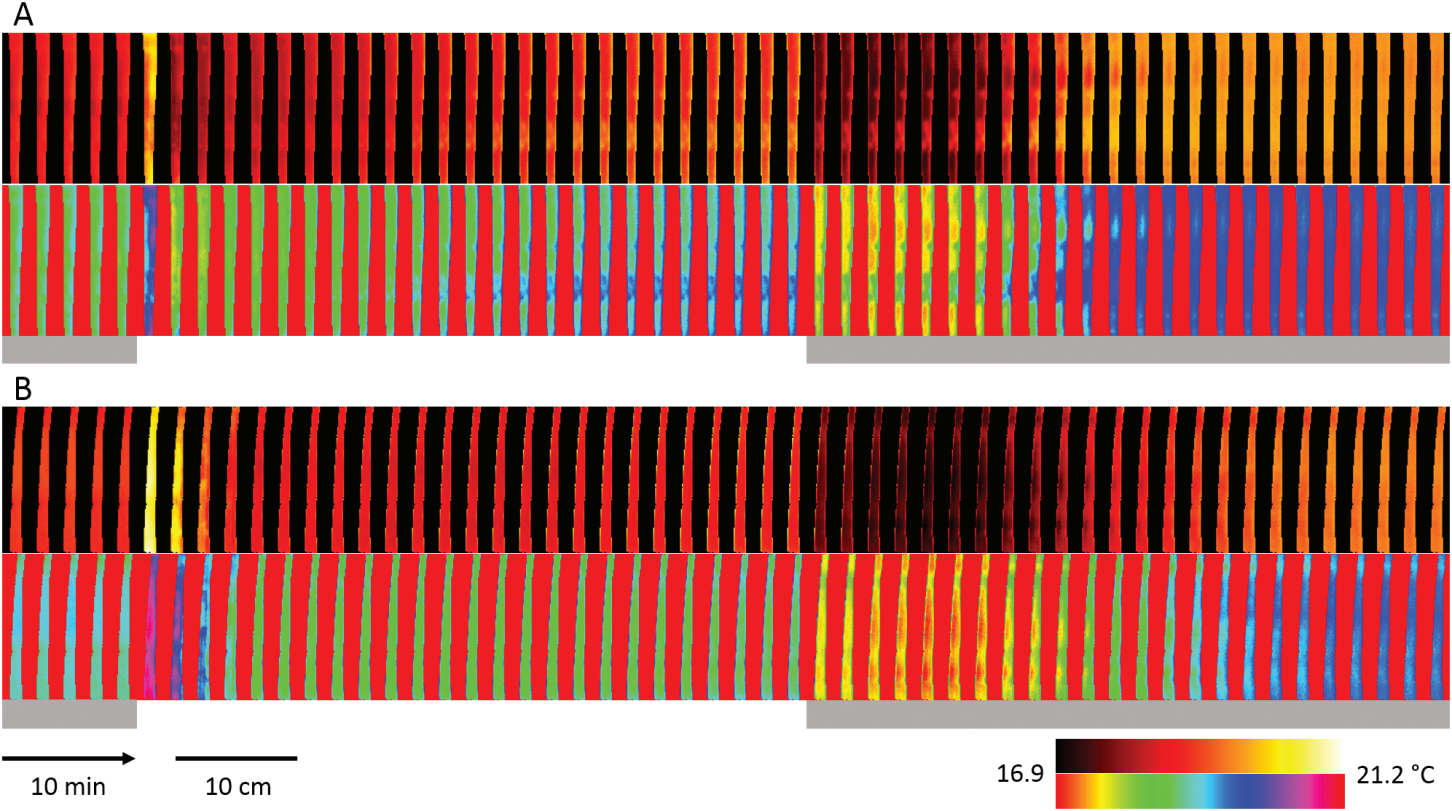
Time-series of thermal images displaying leaf temperature spatiotemporal differences for two leaves (A and B) subjected to changes in light intensities (grey background, 0 μmol m^–2^ s^–1^; white background, 430 μmol m^–2^ s^–1^). Two different colour scales are used to highlight either temperature kinetics or heterogeneity over the leaf surface. Average temperature kinetics for (A) leaf 4 and (B) leaf 5 are visible in Fig. 5

**Fig. 5.**
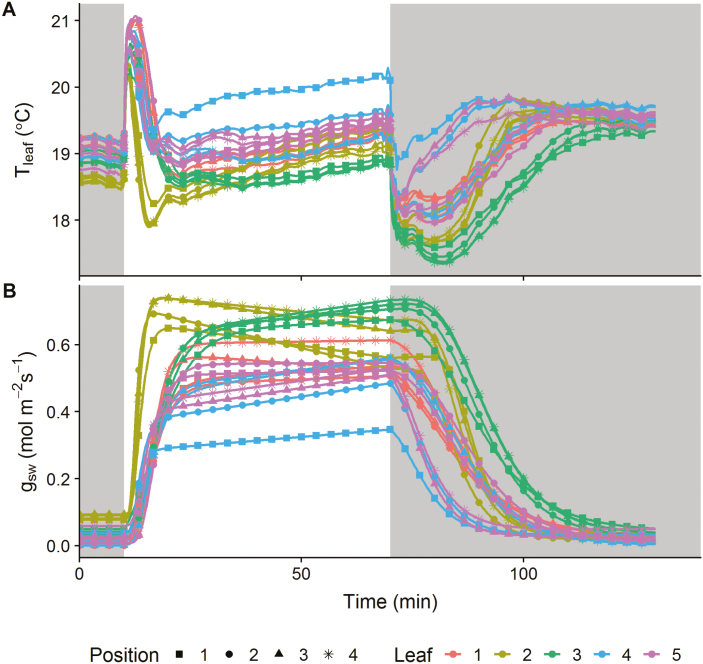
Spatial and temporal response of (A) leaf temperature (*T*_leaf_) and (B) stomatal conductance (*g*_sw_) to step changes in light intensity. Dark areas represent a period where light intensity was 0 µmol m^–2^ s^–1^ and the white area a period where light intensity was 430 µmol m^–2^ s^–1^. Each colour represents a leaf, and each curve represents a different leaf position.

Large spatiotemporal variation in leaf temperature kinetics was observed between and within leaves of wheat (Fig. 4). A false-colour pallet with a continuous gradient in colour (black to white; see Fig. 4) was used to highlight the temporal changes in temperature that were observed following a step increase in light intensity. Both leaves examined behave differently, with areas reaching the highest and lowest temperature at different times within the response. A second false-colour scale separating data into discrete (colour) bands illustrates the large spatial heterogeneity within the leaves and revealed that different parts of the leaf behaved differently over time. To assess the spatial variation in temperature kinetics over the leaf, the leaves were divided into four separate areas from the tip to the bottom of the leaf. The selected areas displayed differences in temperature of between ~0.5 °C and 1.5 °C during the experiment, as well as significant differences in the temporal responses (Fig. 5A). During the initial dark period (first 10 min), leaf temperature differences (as high as 0.7 °C) were mainly influenced by the amount of transpiration, whereas the initial temperature increased when the light was switched on (first 5 min), and depended on light intensity and leaf characteristics (i.e. thickness, density, and specific heat capacity). This increase in temperature was only counterbalanced once stomata started to respond to the light, opening and increasing leaf evaporative cooling by transpiration. Towards the end of the light period, leaf temperature was mainly influence by the surrounding environmental conditions, tracking the decrease in air RH and increase in air temperature that together influence the leaf to air vapour pressure deficit (VPDI; Supplementary Fig. S4). During the second dark period (last hour), the large differences in leaf temperature were due to the level of leaf transpiration achieved during the light period and the rapidity of stomatal closure.

#### Description of stomatal conductance kinetics

Using an energy balance model fitted on the previously described leaf temperature kinetics (Fig. 5A; Supplementary Fig. S5A), *g*_sw_ was derived (Fig. 5B; Supplementary Fig. S5B) precisely for all areas and leaves assessed (Supplementary Fig. S6, rmse 0.069 °C). During the experiment, differences in stomatal behaviour and regulation of transpiration were observed within and between the leaves (Fig. 5B). During the initial dark period, *g*_sw_ values ranged between 0.001 mol m^–2^ s^–1^ and 0.092 mol m^–2^ s^–1^, and at the end of the light period *g*_sw_ ranged between 0.346 mol m^–2^ s^–1^ and 0.733 mol m^–2^ s^–1^, illustrating the large diversity in the regulation of transpiration. During the second dark period, *g*_sw_ continued to increase for some individuals at the beginning and did not return to its original values, displaying a general decrease, with values ranging from 0.004 mol m^–2^ s^–1^ to 0.051 mol m^–2^ s^–1^.

#### Interpretation of stomatal conductance kinetics using an energy balance model

**Fig. 6.**
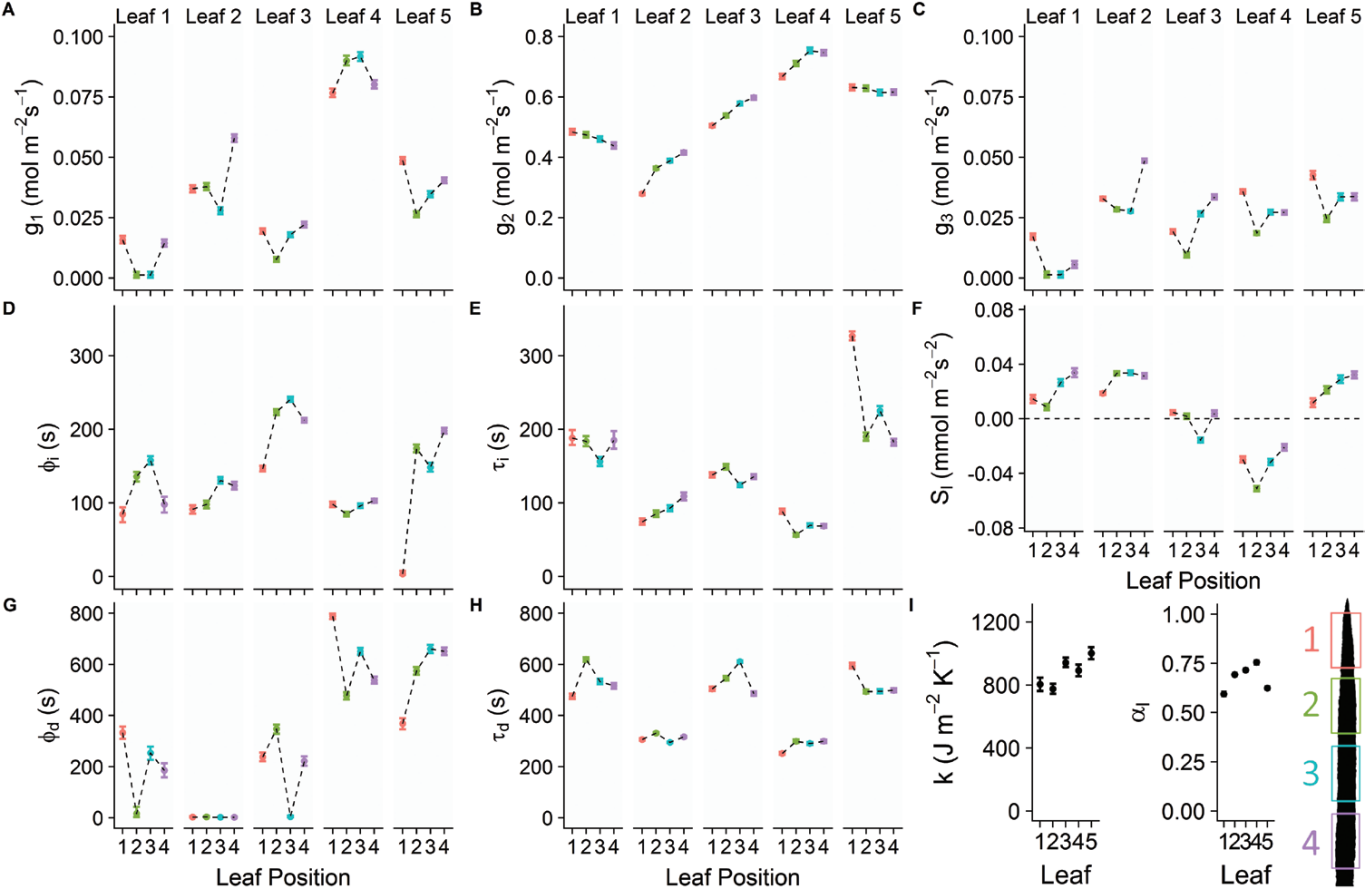
Parameter values (A–K) derived from the leaf energy balance model using Bayesian inference. Steady-state target for the stomatal conductance to water vapour (*g*_sw_, presented on a different scale for dark and light periods) (A) during the initial dark period (*g*_1_), (B) the light period (*g*_2_), and (C) the final dark period (*g*_3_). (D) Time lag and time constant for (D and E) an increase (ϕ_i_ and τ_i_) and (G and H) a decrease (ϕ_d_ and τ_d_) in *g*_sw_. (F) The slow increase or decrease in *g*_sw_ over time (*s*_l_), with the dotted line representing 0. (I) The amount of energy per unit area required to change the temperature of the material by 1 °K (*k*). (J) The leaf shortwave absorbance (α_l_). The model was fitted on observations from five leaves, each divided into four areas (K). Error bars represent the 95% credible interval. Colours represent the different leaf positions.

By fitting the energy balance model on the observed leaf temperature kinetics, parameter values describing the temporal response of *g*_sw_ and the thermal exchanges between the leaf and atmosphere were estimated using Bayesian inference (Fig. 6). Leaf-level boundary layer conductance (*g*_bw_) was estimated at 1.284±0.007SD mol m^–2^ s^–1^ and no significant variation between leaves was observed. Boundary layer conductance values were sufficiently high so as not to be the main limiting process for gas diffusion during the experiment. An important physiological parameter to understand the thermal response of the leaf was the amount of energy per unit area required to change its temperature by 1 °K (*k*) which displayed significant differences but was within a contained range of values (775–1002 J m^–2^ K^–1^). The leaf shortwave absorbance (α_l_) was significantly different between leaves, with values ranging between 0.59 and 0.75, respectively, for leaf 1 and 4. The credible intervals of the estimated parameter were such that it was possible to study the variation of stomatal behaviour not only between leaves but also within individual leaves. Steady-state targets of *g*_sw_ were defined for each dark/light/dark period (*g*_1_, *g*_2_, and *g*_3_) and showed significant variation within and between the leaves (Fig. 6A–C). The initial steady-state *g*_sw_ values during the first dark period (*g*_1_) were significantly different between leaves, with up to 10-fold higher values observed for leaf 4 compared with leaf 1 (Fig. 6A). In the second dark period, values of *g*_3_ showed less variation between individuals, with an average value of ~0.025 mol m^–2^ s^–1^. Spatial differences in *g*_1_ and *g*_3_ of ~0.020 mol m^–2^ s^–1^ were observed within individual leaves. During the light period, *g*_2_ values showed significant differences between leaves (Fig. 6B), including a positive gradient (~0.1 mol m^–2^ s^–1^ increase) along the leaf lamina (between the tip and base of the leaf) for leaves 2–4 and a negative gradient for leaves 1 and 5 (~0.02 mol m^–2^ s^–1^ decrease).

In general, temporal responses of *g*_sw_ displayed an ~2-fold faster increase (Fig. 6D–E) than decrease (Fig. 6G–H), with significant differences observed between leaves. Initial lag time (ϕ_i_ and ϕ_d_) showed greater within-leaf variability than between leaves. Interestingly, leaves with low time constant values for an increase in *g*_sw_ (τ_i_) also showed low time constant values for a decrease in *g*_sw_ (τ_d_). The slow increase or decrease of *g*_sw_ (*S*_l_) observed at the end of the first exponential response after the light was switched on displayed large differences between leaves (Fig. 6F), with positive values (increase) for leaves 1, 2, and 5, and negative values (decrease) for leaf 4. This behaviour was relatively conserved across the leaf lamina.

### Thermal kinetics under rapidly changing environmental conditions

**Fig. 7.**
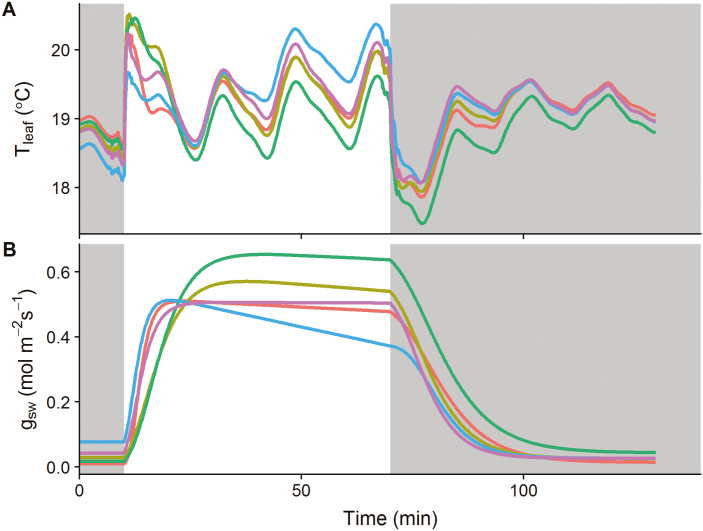
Temporal response of (A) leaf temperature (*T*_leaf_) and (B) stomatal conductance (*g*_sw_) to step changes in light intensity under fluctuating environmental conditions (air temperature and relative humidity). Dark areas represent a period where light intensity was 0 µmol m^–2^ s^–1^ and the white area a period where light intensity was 430 µmol m^–2^ s^–1^. Each colour represents a leaf, and each curve represents a leaf section.

**Fig. 8.**
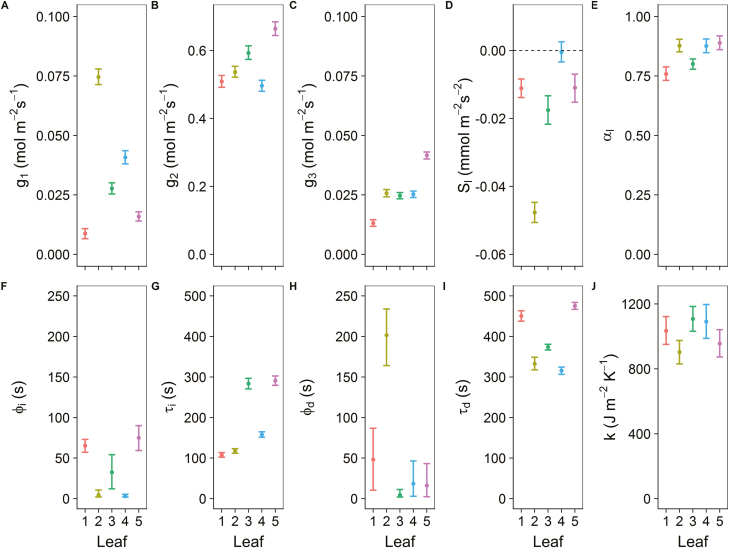
Parameter values (A–J) derived from the leaf energy balance model using Bayesian inference. Steady-state target for the stomatal conductance to water vapour (*g*_sw_) (A) during the initial dark period (*g*_1_), (B) the light period (*g*_2_), and (C) the final dark period (*g*_3_). (D) The slow increase or decrease in *g*_sw_ over time (*s*_l_), with the dotted line representing 0. (E) The leaf shortwave absorbance (α_l_). The time lag and the time constant for (F and G) an increase (ϕ_i_ and τ_i_) and (H and I) a decrease (ϕ_d_ and τ_d_) in *g*_sw_. (J) The amount of energy per unit area required to change the temperature of the material by 1 °K (*k*). The model was fitted on observations from five leaves. Error bars represent the 95% credible interval. Colours represent the different leaf areas.

To illustrate the robustness of the proposed model to variation in environmental conditions, five leaves were assessed under fluctuating air temperature and relative humidity (Supplementary Fig. S7) to produce complex leaf evaporative demand and temperature kinetics (Fig. 7A). Despite the more complex leaf temperature fluctuations and the fact that the *g*_sw_ model was developed to consider mainly variation in light intensity, model predictions showed a similar precision (Supplementary Fig. S8, rmse 0.098 °C) to the predicted leaf temperature kinetics previously described in Fig. 5 (Supplementary Fig. S6, rmse 0.069 °C). Estimation of *g*_sw_ showed a rapid increase after the light was switched on, followed by a plateau or a slow decrease, and a relatively slow decrease of *g*_sw_ when the light was switched off (Fig. 7B). In general, parameter values derived from the energy balance model in Fig. 8 were within the same range as those observed previously in Fig. 6. The steady-state targets for *g*_sw_ for each period of the kinetics (*g*_1_, *g*_2_, and *g*_3_) showed no consistent pattern but significant differences between leaves. All leaves presented a slow decrease in *g*_sw_ after the initial exponential increase, with one leaf exhibiting a 4-fold lower value of *s*_l_. Shortwave absorbances were slightly higher than those displayed in Fig. 6, with values ranging from 0.76 to 0.89. Time constants for an increase in *g*_sw_ (τ_i_) were about half the values of those estimated for a decrease in *g*_sw_ (τ_d_), with significant differences between leaves. Lag times (ϕ) for the increase or the decrease of *g*_sw_ had a similar magnitude in both cases. The amount of energy per unit area required to change the temperature of the material by 1 °K (*k*) was only significantly different between leaf 2 and 3, with an average for all leaves of 1018 J m^–2^ K^–1^ (Fig. 8J).

### Parameter importance for dynamic energy balance predictions

**Fig. 9.**
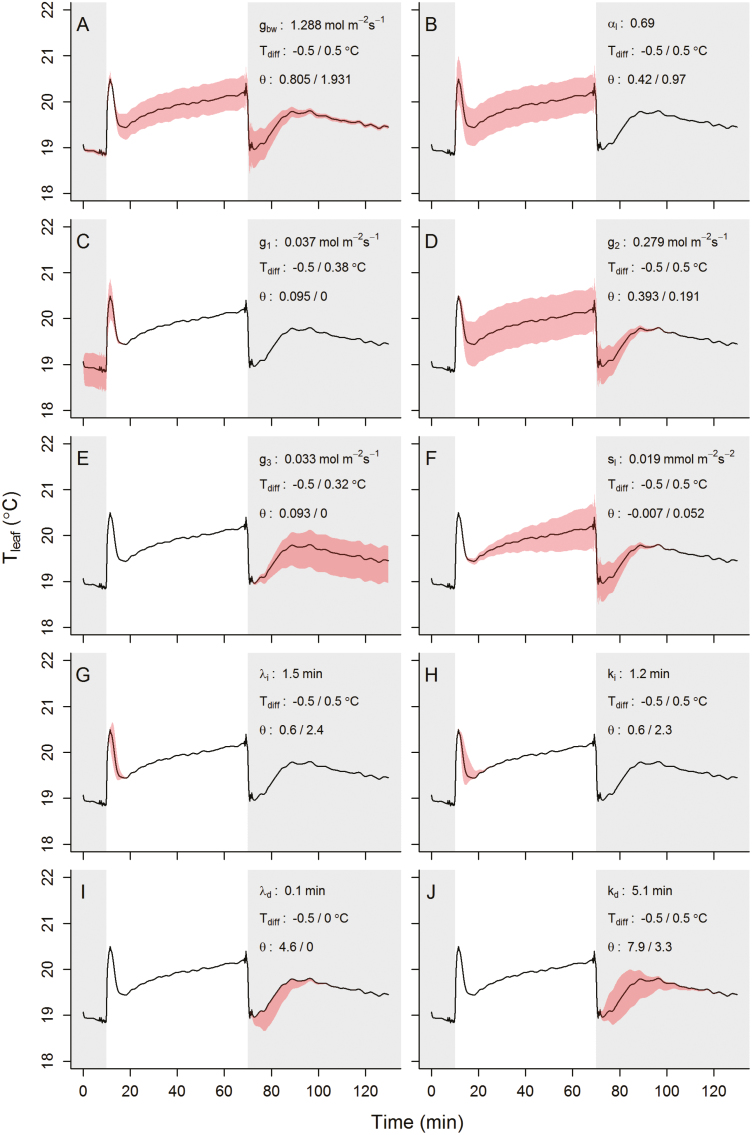
Sensitivity analysis representing the variation of parameter values required to change leaf temperature by ±0.5 °C during step changes of light intensity. Parameter values from leaf 2 and area 1 were used as an illustration (Fig. 5). Dark areas represent a period where light intensity was 0 µmol m^–2^ s^–1^ and the white area a period where light intensity was 430 µmol m^–2^ s^–1^. Differences in leaf temperature were only achieved over parts of the temperature kinetics depending on the parameter (red shaded area) and reached during these periods a maximum of ±0.5 °C. Original parameter values and corresponding curves (solid black line) are displayed in each plot, with the achieved temperature differences (*T*_diff_) and the corresponding parameter values (θ). In some cases (C, E, F, G, I), the maximum temperature deviation of ±0.5 °C was not reached due to a parameter value reaching a boundary (e.g. 0); the value achieved was shown instead of ±0.5 °C.

Under dynamic environmental conditions, variations in parameter values in the energy balance equations impact leaf temperature kinetics with a different magnitude at different periods throughout the kinetics. The impact of these values on temperature kinetics is displayed in Fig. 8, and illustrates how much parameter values need to be adjusted to achieve a ±0.5 °C temperature variation within the step changes in light intensity. The results illustrated the relative importance of the boundary layer conductance (*g*_bw_, Fig. 9A), which influenced the entire temporal response of leaf temperature when changed by about ±50%. Leaf shortwave absorbance (α_l_) impacted leaf temperature during the light period but required variations in value that were biologically improbable, as alterations of 50% were required to drive a 0.5 °C change in temperature. Steady-state targets for *g*_sw_ (*g*_1_, *g*_2_, and *g*_3_) impacted leaf temperature kinetics during the period for which they are defined as well as the initial part of the following period, with variations that were within the observed range of values shown in Figs 6 and 8. During the light period, increasing the leaf temperature by 0.5 °C required a 3-fold increase in the value of the slow increase or decrease in *g*_sw_ (*s*_l_). Parameters influencing the temporal response of *g*_sw_ (Fig. 9G–J) only had a transient impact on leaf temperature but did not require large variations in values. For example, increasing the initial time lag (ϕ_*i*_) or the time constant (τ_i_) by ~1 min after a step increase in light intensity resulted in a 0.5 °C increase in leaf temperature, which lasted for 10 min (Fig. 9G, H). During the second dark period, increasing the initial time lag (ϕ_*d*_) or the time constant (τ_d_) by ~3 min resulted in a 0.5 °C decrease in leaf temperature, which lasted for at least 30 min (Fig. 9I, J).

## Discussion

Despite the success of thermometry in selecting plants with improved yield or altered response to drought (Raskin and Ladyman, 1988; Reynolds *et al.*, 1999; Merlot *et al.*, 2002), a major limitation of this technique is the need for stable environmental conditions to interpret the temperature differences (Reynolds *et al.*, 1999; Jones *et al.*, 2009; Rischbeck *et al.*, 2017; Prado *et al.*, 2018).The results presented here provide strong evidence that thermography can be used to derive *g*_sw_ under a dynamic environment, opening up a new avenue for plant phenotyping and selection. This is particularly relevant given the growing evidence that temporal responses of *g*_sw_ limit photosynthesis and potentially impact yield (McAusland *et al.*, 2016; Taylor and Long, 2017; Vialet-Chabrand *et al.*, 2017*b*).

Our approach to describe the combination of leaf energy balance and mass transfer does not depend on any specific reference material or environmental conditions, making this method a versatile tool for thermography. The equations described in this study have been applied using aluminium references to predict, with high accuracy, leaf temperature kinetics in wheat, allowing *g*_sw_ to be derived under a fluctuating light environment. Model outputs were validated using a leaf replica with a known conductance to water vapor subjected to the same fluctuating conditions. Although leaf replicas have been used in the past to study the importance of mass transfer in leaf energy balance (Zwieniecki *et al.*, 2016; Schymanski and Or, 2017; Schymanski *et al.*, 2017), our results highlight the potential of the low-cost leaf replica and references to validate leaf temperature predictions obtained using energy balance equations. The ability to derive *g*_sw_ from thermography under a fluctuating environment using a relatively simple set-up opens the way to field measurements in the future, but will require further improvements to take into consideration parameters such as leaf orientations and local variations in *L*_d_. In this context, our approach could be combined with hemispherical references placed at different positions in the field (Jones *et al.*, 2018) to take into account the different leaf angles and surrounding thermal conditions present in a field canopy. Indeed, our model can be easily adapted to an existing experimental set-up or used to reprocess existing data if enough information is available. Previous work has attempted to use dynamic energy balance equations (taking into consideration the temporal dimension) but are often incomplete in their implementation or use approximations to describe the continuous interactions between the leaf and a fluctuating environment (Bajons *et al.*, 2005; Schymanski *et al.*, 2013; Page *et al.*, 2018). The ability of the model presented here to operate under any environmental condition is mainly due to the unique combination of equations integrating the continuous variations of the environmental variables, the impact of stomatal behaviour, and ultimately leaf temperature.

Combining stomatal conductance and energy balance models opens up new possibilities to use leaf temperature kinetics to improve our understanding of the mechanisms involved in stomatal responses to the surrounding environment. However, combining both models requires an estimate of boundary layer conductance (*g*_bw_) that dictates part of the gas and heat exchange. Therefore, our model simultaneously estimates *g*_sw_, *g*_bw_, and leaf temperature in fluctuating environmental conditions using a set-up that is easy to use and low cost, while providing precise estimates. The values of *g*_bw_ used to calculate *g*_sw_ from the energy balance equations produced *g*_sw_ values that were close to those of the leaf replica, suggesting that our estimates of *g*_bw_ were accurate enough to derive a valid *g*_sw_ value. Numerous research studies has been published on measuring leaf boundary layer conductance (Leuning *et al.*, 1989; Leuning, 1990; Brenner and Jarvis, 1995; Stokes *et al.*, 2006; Katsoulas *et al.*, 2007) and although our method is derived from a similar theoretical basis, it does not require specific equipment (e.g. power source, heating element) and relies on environmental variations, making it simple to operate. In the case of portable gas exchange chambers, investigating stomatal responses requires artificial conditions that alter the thermal exchange between leaf and atmosphere (e.g. influence of wall temperature) and often use high boundary layer conductance to simplify the measurements. Using thermography enables the determination of *g*_sw_ without artificially altering the conditions surrounding the leaf, meaning that measurements of leaf responses are closer to those observed in the field. The outcomes from our modelling approach included the temporal response of *g*_sw_, a biological trait that has attracted significant attention in recent years (Lawson *et al.*, 2010; Lawson and Blatt, 2014; McAusland *et al.*, 2016; Meinzer *et al.*, 2017; Matthews *et al.*, 2018; Deans *et al.*, 2018) because of its impact on both photosynthesis and water use efficiency.

Nocturnal stomatal conductance (observed after dark acclimation) was significantly different between individuals, suggesting differences in the regulation of water loss during the night period. Nocturnal transpiration is involved in essential physiological processes such as nutrient transport (Zeppel *et al.*, 2014) and could represent 30% of daytime water consumption in crops (Claverie *et al.*, 2018). The initial *g*_sw_ values observed here under dark conditions represented up to ~10% of the value reached under the light period, confirming its importance for the regulation of plant water budget. Nocturnal regulation of *g*_sw_ has been shown to be developmentally and genetically controlled in wheat (Claverie *et al.*, 2016; Schoppach *et al.*, 2016) and has been proposed as a breeding target to produce plants with improved tolerance to drought (Schoppach *et al*., 2014, 2016; Coupel-Ledru *et al.*, 2016). These results highlight the potential of our method for future breeding programmes aiming to screen populations with differences in nocturnal transpiration.

Under the light period, *g*_sw_ values were similar to those reported previously in the literature for wheat (McAusland *et al.*, 2016; Taylor and Long, 2017). However, stomatal responses reported here were more rapid than those reported for wheat by McAusland *et al.* (2016) and displayed a slow decrease in *g*_sw_ after the initial exponential response, which was also significantly different between leaves. These two independent estimations of the temporal response of *g*_sw_ for wheat were performed under different environmental conditions (controlled versus uncontrolled) and we believe that the rapid increase of leaf temperature after the light was switched on (due to the increase in incident energy), simultaneously with a decrease in air RH and an increase in air temperature experienced by the whole plant in the imaging area, may have contributed to this change in stomatal behaviour. Interestingly, Franks and Farquhar (2007) observed similar enhancement in wheat of the initial rate of increase in *g*_sw_ and a lower final value at high VPDI (2 kPa) compared with low VPDI (1 kPa). Mott *et al.* (1999) proposed that the difference in response depends on hydraulic interactions among stomata that are mediated by transpiration-induced changes in epidermal turgor, which could explain our observations. In addition, after a step increase in light intensity, we observed an initial lag (~2–3 min) before *g*_sw_ changed significantly, confirming the observations in wheat made by Taylor and Long (2017). This initial lag has been shown to impact photosynthesis in different species (Vialet-Chabrand *et al.*, 2013; McAusland *et al.*, 2016; Taylor and Long, 2017) but also leads to a rapid increase in leaf temperature due to the limited cooling by transpiration. Schymanski *et al.* (2013) highlighted the potential heat damage and hydraulic failure a leaf may experience during the first minutes of a sun fleck, which could be avoided by maintaining *g*_sw_ at a relatively high value when the leaf returns to shade conditions. During our experiment, the lag time and rapidity of response for a decrease in *g*_sw_ were substantially larger than for an increase in *g*_sw_, ensuring that *g*_sw_ remained at a high value for a longer time period. Additionally, the sensitivity analysis performed on the model revealed that a small increase in parameter values ϕ_i/d_ and τ_i/d_, controlling the rapidity of *g*_sw_ variation, resulted in maintaining a low leaf temperature for a greater duration after a decrease in light intensity. These observations are compatible with a conservative strategy to maximize carbon fixation by unit of water loss (Ooba and Takahashi, 2003) and limit heat damage (Schymanski *et al.*, 2013) under a fluctuating light environment. Even if there is asymmetry in the response between the rapidity of increase and decrease in *g*_sw_, it is interesting to note that leaves with a slow increase in *g*_sw_ also had a slow decrease, suggesting a co-ordination of both responses as proposed by McAusland *et al.* (2016). Overall, the diversity observed here for only a few individuals is a promising result that could be used as breeding targets to improve yield by reducing the stomatal limitation on photosynthesis as suggested by Lawson and Blatt (2014), as well as to enhance thermal regulation and water-saving strategies to limit heat damage and increase drought tolerance in hot and dry environments.

Under a dynamic environment, thermograms revealed variation in temperature kinetics over the leaf lamina that could be attributed to patchy stomatal behaviour (Weyers and Lawson, 1997; Lawson and Weyers, 1999). Compared with previous research studying the spatial heterogeneity of *g*_sw_ using thermograms (Jones, 1999; Saudreau *et al.*, 2017; Sweet *et al.*, 2017; Page *et al.*, 2018), we assessed the temporal response of *g*_sw_ for different sections of wheat leaves following a step increase in light intensity to characterize the rapidity and the magnitude of the stomatal response. The spatial gradient observed for the magnitude of the *g*_sw_ response (up to an ~50% increase from the base to the tip of the leaf) stressed the importance of measuring *g*_sw_ at a similar leaf position for each individual when using a portable gas exchange chamber (Lawson and Weyers, 1999). Estimations of the rapidity of the stomatal response are less influenced by the position on the leaf blade, suggesting that at this scale in wheat, any patchy effect is mainly due to variation of stomatal density rather than different stomatal behaviour. Prytz *et al.* (2003) monitored spatial and temporal stomatal conductance of *Avena* subject to low humidity and suggested that the synchronicity of stomatal behaviour at different leaf positions may be a result of the anatomy of monocotyledonous leaves in which the main veins running parallel with the leaf blade transport a hydraulic signal synchronizing different areas of the leaf in the longitudinal direction. More specifically, in wheat, Buckley and Mott (2000) provided strong evidence for long‐distance hydraulic interactions co-ordinating the stomatal response in different areas of the leaf that could explain our observations. Previous research using infrared gas exchange to measure stomatal behaviour has shown that the temporal response of *g*_sw_ displayed significant variations within (Matthews *et al.*, 2018) and between species (McAusland *et al.*, 2016) with comparable responses to our results. Previous studies using thermal signatures to assess stomatal kinetics have been limited to the first few minutes of a response to a step change in light intensity (Bajons *et al.*, 2005; Page *et al.*, 2018) or have only interpreted the relative changes in temperature (Prytz *et al.*, 2003). Overall, the model presented here allowed us to investigate the parameters controlling the temporal response of *g*_sw_ (τ, ϕ, *S*_l_) that have been related to guard cell metabolism (Hills *et al.*, 2012; Wang *et al.*, 2014; Vialet-Chabrand *et al.*, 2017*a*), and provided strong evidence of a co-ordinated rapidity of response across the leaf lamina in wheat despite the local variation in the magnitude of the response.

The findings and experimental approaches presented here have the potential to remove a major bottleneck in high-throughput phenotyping of stomatal-related traits, by allowing the interpretation of thermograms under a fluctuating environment. Time-series of thermograms associated with our new energy balance approach provided spatial and temporal characterization of stomatal conductance (*g*_sw_) responses in wheat, highlighting the importance of co-ordinated stomatal responses across the leaf blade. The diversity and asymmetry of the temporal response of *g*_sw_ observed after a step increase or decrease in light intensity can be interpreted as a strategy to maximize photosynthesis per unit of water loss and avoid heat stress under a fluctuating environment. Improving these aspects of stomatal behaviour is thought to be an important breeding target for future yield improvement, to which our work will directly contribute by allowing screening of large numbers of plants for biologically relevant traits. The techniques and methods presented here provide significant evidence for the accuracy of predicted *g*_sw_ from leaf temperature kinetics, that can be easily transferred to existing equipment and will pave the way for further development in our understanding of stomatal behaviour in future field studies.

## Supplementary data

Supplementary data are available at *JXB* online.

**Table S1.** Equations required to derive environmental variables from the raw environmental measurements collected from various sensors.

**Fig. S1.** Example of signal processing to remove high-frequency noise from infrared thermal measurement.

**Fig. S2.** Example of temperature kinetics measured using thermal imaging.

**Fig. S3.** Comparison of temperature measurements using an infrared thermal camera and a thermocouple.

**Fig. S4.** Environmental conditions during step changes of light intensity.

**Fig. S5.** Example of the performance of the energy balance model to reproduce leaf temperature kinetics and stomatal conductance.

**Fig. S6.** Performance of the energy balance model to reproduce leaf temperature kinetics.

**Fig. S7.** Environmental conditions during step changes of light intensity.

**Fig. S8.** Performance of the energy balance model to reproduce leaf temperature kinetics.

**Algorithm S1.** Equations describing the energy balance model.

Supplementary Table S1 and Figures S1-S8 and Protocol_S1

## References

[CIT0001] Bajons P, Klinger G, Schlosser V 2005 Determination of stomatal conductance by means of infrared thermography. Infrared Physics and Technology 46, 429–439.

[CIT0002] Brenner AJ, Jarvis PG 1995 A heated leaf replica technique for determination of leaf boundary layer conductance in the field. Agricultural and Forest Meteorology 72, 261–275.

[CIT0003] Brown HT, Escombe F 1900 Static diffusion of gases and liquids in relation to the assimilation of carbon and translocation in plants. Philosophical Transactions of the Royal Society B: Biological Sciences 193, 223–291.

[CIT0004] Buckley TN, Mott KA 2000 Stomatal responses to non-local changes in PFD: evidence for long-distance hydraulic interactions. Plant, Cell & Environment 23, 301–309.

[CIT0005] Carpenter B, Gelman A, Hoffman MD, Lee D, Goodrich B, Betancourt M, Brubaker M, Guo J, Li P, Riddell A 2017 Stan : a probabilistic programming language. Journal of Statistical Software 76, doi: 10.18637/jss.v076.i01. PMC9788645 36568334

[CIT0006] Claverie E, Meunier F, Javaux M, Sadok W 2018 Increased contribution of wheat nocturnal transpiration to daily water use under drought. Physiologia Plantarum 162, 290–300. 28833246 10.1111/ppl.12623

[CIT0007] Claverie E, Schoppach R, Sadok W 2016 Nighttime evaporative demand induces plasticity in leaf and root hydraulic traits. Physiologia Plantarum 158, 402–413. 27235372 10.1111/ppl.12474

[CIT0008] Costa JM, Monnet F, Jannaud D, Leonhardt N, Ksas B, Reiter IM, Pantin F, Genty B 2015 Open all night long: the dark side of stomatal control. Plant Physiology 167, 289–294. 25527716 10.1104/pp.114.253369 PMC4326751

[CIT0009] Coupel-Ledru A, Lebon E, Christophe A, Gallo A, Gago P, Pantin F, Doligez A, Simonneau T 2016 Reduced nighttime transpiration is a relevant breeding target for high water-use efficiency in grapevine. Proceedings of the National Academy of Sciences, USA 113, 8963–8968. 10.1073/pnas.1600826113 PMC4987834 27457942

[CIT0010] Cowan IR 1978 Stomatal behaviour and environment. Advances in Botanical Research 4, 117–228.

[CIT0011] Deans RM, Brodribb TJ, Busch FA, Farquhar GD 2018 Plant water-use strategy mediates stomatal effects on the light induction of photosynthesis. New Phytologist doi: 10.1111/nph.15572. 30372523

[CIT0012] Fahlgren N, Gehan MA, Baxter I 2015 Lights, camera, action: high-throughput plant phenotyping is ready for a close-up. Current Opinion in Plant Biology 24, 93–99. 25733069 10.1016/j.pbi.2015.02.006

[CIT0013] **FAO, IFAD, UNICEF, WFP, WHO** 2018 The state of food security and nutrition in the world 2018. Building climate resilience for food security and nutrition. Rome: FAO.

[CIT0014] Fiorani F, Schurr U 2013 Future scenarios for plant phenotyping. Annual Review of Plant Biology 64, 267–291. 10.1146/annurev-arplant-050312-120137 23451789

[CIT0015] Fischer RA, Edmeades GO 2010 Breeding and cereal yield progress. Crop Science 50, S85–S98.

[CIT0016] Fischer RA, Rebetzke GJ 2018 Indirect selection for potential yield in early-generation, spaced plantings of wheat and other small-grain cereals: a review. Crop and Pasture Science 69, 439–459.

[CIT0017] Franks PJ, Farquhar GD 2007 The mechanical diversity of stomata and its significance in gas-exchange control. Plant Physiology 143, 78–87. 17114276 10.1104/pp.106.089367 PMC1761988

[CIT0018] Furbank RT, Tester M 2011 Phenomics—technologies to relieve the phenotyping bottleneck. Trends in Plant Science 16, 635–644. 22074787 10.1016/j.tplants.2011.09.005

[CIT0019] Ghanem ME, Marrou H, Sinclair TR 2015 Physiological phenotyping of plants for crop improvement. Trends in Plant Science 20, 139–144. 25524213 10.1016/j.tplants.2014.11.006

[CIT0020] Guilioni L, Jones HG, Leinonen I, Lhomme JP 2008 On the relationships between stomatal resistance and leaf temperatures in thermography. Agricultural and Forest Meteorology 148, 1908–1912.

[CIT0021] Haudry A, Cenci A, Ravel C, et al 2007 Grinding up wheat: a massive loss of nucleotide diversity since domestication. Molecular Biology and Evolution 24, 1506–1517. 17443011 10.1093/molbev/msm077

[CIT0022] Hills A, Chen ZH, Amtmann A, Blatt MR, Lew VL 2012 OnGuard, a computational platform for quantitative kinetic modeling of guard cell physiology. Plant Physiology 159, 1026–1042. 22635116 10.1104/pp.112.197244 PMC3387691

[CIT0023] Jones HG 1999 Use of thermography for quantitative studies of spatial and temporal variation of stomatal conductance over leaf surfaces. Plant, Cell & Environment 22, 1043–1055.

[CIT0024] Jones HG 2013 Plants and microclimate. Cambridge: Cambridge University Press.

[CIT0025] Jones HG, Hutchinson PA, May T, Jamali H, Deery DM 2018 A practical method using a network of fixed infrared sensors for estimating crop canopy conductance and evaporation rate. Biosystems Engineering 165, 59–69.

[CIT0026] Jones HG, Leinonen I 2003 Thermal imaging for study of plant water relations. Agricultural Meteorology 59, 205–217.

[CIT0027] Jones HG, Serraj R, Loveys BR, Xiong L, Wheaton A, Price AH 2009 Thermal infrared imaging of crop canopies for the remote diagnosis and quantification of plant responses to water stress in the field. Functional Plant Biology 36, 978–989. 10.1071/FP09123 32688709

[CIT0028] Katsoulas N, Baille A, Kittas C 2007 Leaf boundary layer conductance in ventilated greenhouses: an experimental approach. Agricultural and Forest Meteorology 144, 180–192.

[CIT0029] Lawson T, Blatt MR 2014 Stomatal size, speed, and responsiveness impact on photosynthesis and water use efficiency. Plant Physiology 164, 1556–1570. 24578506 10.1104/pp.114.237107 PMC3982722

[CIT0030] Lawson T, von Caemmerer S, Baroli I 2010 Photosynthesis and stomatal behaviour. In: Lüttge UE, Beyschlag W, Büdel B, Francis D, eds. Progress in botany. Berlin, Heidelberg: Springer, 265–304.

[CIT0031] Lawson T, Weyers J 1999 Spatial and temporal variation in gas exchange over the lower surface of *Phaseolus vulgaris* L. primary leaves. Journal of Experimental Botany 50, 1381–1391.

[CIT0032] Lehmann P, Or D 2015 Effects of stomata clustering on leaf gas exchange. New Phytologist 207, 1015–1025. 25967110 10.1111/nph.13442

[CIT0033] Leinonen I, Grant OM, Tagliavia CP, Chaves MM, Jones HG 2006 Estimating stomatal conductance with thermal imagery. Plant, Cell & Environment 29, 1508–1518. 10.1111/j.1365-3040.2006.01528.x 16898014

[CIT0034] Leuning R 1990 Modelling stomatal behaviour and and photosynthesis of *Eucalyptus grandis*. Australian Journal of Plant Physiology 17, 159–175.

[CIT0035] Leuning R, Foster IJ 1990 Estimation of transpiration by single trees: comparison of a ventilated chamber, leaf energy budgets and a combination equation. Agricultural and Forest Meteorology 51, 63–86.

[CIT0036] Leuning R, Grace J, Monteith JL, Milford JR, Unsworth MH, Fowler D 1989 Leaf energy balances: developments and applications [and discussion]. Philosophical Transactions of the Royal Society B: Biological Sciences 324, 191–206.

[CIT0037] Li L, Zhang Q, Huang D 2014 A review of imaging techniques for plant phenotyping. Sensors 14, 20078–20111. 25347588 10.3390/s141120078 PMC4279472

[CIT0038] Long SP, Marshall-Colon A, Zhu XG 2015 Meeting the global food demand of the future by engineering crop photosynthesis and yield potential. Cell 161, 56–66. 25815985 10.1016/j.cell.2015.03.019

[CIT0039] Maes WH, Baert A, Huete AR, Minchin PEH, Snelgar WP, Steppe K 2016 A new wet reference target method for continuous infrared thermography of vegetations. Agricultural and Forest Meteorology 226–227, 119–131.

[CIT0040] Matthews JSA, Vialet-Chabrand S, Lawson T 2018 Acclimation to fluctuating light impacts the rapidity of response and diurnal rhythm of stomatal conductance. Plant Physiology 176, 1939–1951. 29371250 10.1104/pp.17.01809 PMC5841698

[CIT0041] McAusland L, Davey PA, Kanwal N, Baker NR, Lawson T 2013 A novel system for spatial and temporal imaging of intrinsic plant water use efficiency. Journal of Experimental Botany 64, 4993–5007. 24043857 10.1093/jxb/ert288 PMC3830482

[CIT0042] McAusland L, Vialet-Chabrand S, Davey P, Baker NR, Brendel O, Lawson T 2016 Effects of kinetics of light-induced stomatal responses on photosynthesis and water-use efficiency. New Phytologist 211, 1209–1220. 27214387 10.1111/nph.14000 PMC4982059

[CIT0043] McCouch S 2013 Comment: feeding the future. Nature 499, 23–24. 23823779 10.1038/499023a

[CIT0044] Meinzer FC, Smith DD, Woodruff DR, Marias DE, McCulloh KA, Howard AR, Magedman AL 2017 Stomatal kinetics and photosynthetic gas exchange along a continuum of isohydric to anisohydric regulation of plant water status. Plant, Cell & Environment 40, 1618–1628. 10.1111/pce.12970 28426140

[CIT0045] Merlot S, Mustilli AC, Genty B, North H, Lefebvre V, Sotta B, Vavasseur A, Giraudat J 2002 Use of infrared thermal imaging to isolate *Arabidopsis* mutants defective in stomatal regulation. The Plant Journal 30, 601–609. 12047634 10.1046/j.1365-313x.2002.01322.x

[CIT0046] Monteith JL 1965 Evaporation and environment. Symposia of the Society for Experimental Biology 19, 205–234. 5321565

[CIT0047] Monteith JL, Unsworth MH 2012 Principles of environmental physics: plants, animals, and the atmosphere. Amsterdam: Elsevier.

[CIT0048] Mott KA, Shope JC, Buckley TN 1999 Effects of humidity on light-induced stomatal opening: evidence for hydraulic coupling among stomata. Journal of Experimental Botany 50, 1207–1213.

[CIT0049] Munns R, James RA, Sirault XR, Furbank RT, Jones HG 2010 New phenotyping methods for screening wheat and barley for beneficial responses to water deficit. Journal of Experimental Botany 61, 3499–3507. 20605897 10.1093/jxb/erq199

[CIT0050] Olbrycht R, Więcek B 2015 New approach to thermal drift correction in microbolometer thermal cameras. Quantitative InfraRed Thermography Journal 12, 184–195.

[CIT0051] Ooba M, Takahashi H 2003 Effect of asymmetric stomatal response on gas-exchange dynamics. Ecological Modelling 164, 65–82.

[CIT0052] Page GFM, Liénard JF, Pruett MJ, Moffett KB 2018 Spatiotemporal dynamics of leaf transpiration quantified with time-series thermal imaging. Agricultural and Forest Meteorology 256–257, 304–314.

[CIT0053] Pearcy RW, Way DA 2012 Two decades of sunfleck research: looking back to move forward. Tree Physiology 32, 1059–1061. 22977203 10.1093/treephys/tps084

[CIT0054] Penman HL 1948 Natural evaporation from open water, bare soil and grass. Proceedings of the Royal Society A: Mathematical, Physical and Engineering Sciences 193, 120–145. 10.1098/rspa.1948.0037 18865817

[CIT0055] Pingali PL 2012 Green revolution: impacts, limits, and the path ahead. Proceedings of the National Academy of Sciences, USA 109, 12302–12308. 10.1073/pnas.0912953109 PMC3411969 22826253

[CIT0056] Prado SA, Cabrera-Bosquet L, Grau A, Coupel-Ledru A, Millet EJ, Welcker C, Tardieu F 2018 Phenomics allows identification of genomic regions affecting maize stomatal conductance with conditional effects of water deficit and evaporative demand. Plant, Cell & Environment 41, 314–326. 10.1111/pce.13083 29044609

[CIT0057] Prashar A, Jones H 2014 Infra-red thermography as a high-throughput tool for field phenotyping. Agronomy 4, 397–417. 10.1371/journal.pone.0065816 PMC3676367 23762433

[CIT0058] Prytz G, Futsaether CM, Johnsson A 2003 Thermography studies of the spatial and temporal variability in stomatal conductance of Avena leaves during stable and oscillatory transpiration. New Phytologist 158, 249–258.

[CIT0059] Rahaman MM, Chen D, Gillani Z, Klukas C, Chen M 2015 Advanced phenotyping and phenotype data analysis for the study of plant growth and development. Frontiers in Plant Science 6, 619. 26322060 10.3389/fpls.2015.00619 PMC4530591

[CIT0060] Ramankutty N, Mehrabi Z, Waha K, Jarvis L, Kremen C, Herrero M, Rieseberg LH 2018 Trends in global agricultural land use: implications for environmental health and food security. Annual Review of Plant Biology 69, 789–815. 10.1146/annurev-arplant-042817-040256 29489395

[CIT0061] Raskin I, Ladyman JA 1988 Isolation and characterization of a barley mutant with abscisic-acid-insensitive stomata. Planta 173, 73–78. 24226182 10.1007/BF00394490

[CIT0062] Ray DK, Mueller ND, West PC, Foley JA 2013 Yield trends are insufficient to double global crop production by 2050. PLoS One 8, e66428. 23840465 10.1371/journal.pone.0066428 PMC3686737

[CIT0063] Ray DK, Ramankutty N, Mueller ND, West PC, Foley JA 2012 Recent patterns of crop yield growth and stagnation. Nature Communications 3, 1293. 10.1038/ncomms2296 23250423

[CIT0064] Rebetzke GJ, Condon AG, Richards RA, Read JJ 2001 Phenotypic variation and sampling for leaf conductance in wheat (*Triticum aestivum* L.) breeding populations. Euphytica 121, 335–341.

[CIT0065] Reynolds D, Baret F, Welcker C, et al 2018 What is cost-efficient phenotyping? Optimizing costs for different scenarios. Plant Science doi: 10.1016/j.plantsci.2018.06.015. 31003607

[CIT0066] Reynolds MP, Rajaram S, Sayre KD 1999 Period and approaches for meeting projected global demand. Crop Science 39, 1611–1621.

[CIT0067] Richards RA 2000 Selectable traits to increase crop photosynthesis and yield of grain crops. Journal of Experimental Botany 51 Spec No, 447–458. 10938853 10.1093/jexbot/51.suppl_1.447

[CIT0068] Rischbeck P, Cardellach P, Mistele B, Schmidhalter U 2017 Thermal phenotyping of stomatal sensitivity in spring barley. Journal of Agronomy and Crop Science 203, 483–493.

[CIT0069] Saudreau M, Ezanic A, Adam B, Caillon R, Walser P, Pincebourde S 2017 Temperature heterogeneity over leaf surfaces: the contribution of the lamina microtopography. Plant, Cell & Environment 40, 2174–2188. 10.1111/pce.13026 28710812

[CIT0070] Schoppach R, Claverie E, Sadok W 2014 Genotype-dependent influence of night-time vapour pressure deficit on night-time transpiration and daytime gas exchange in wheat. Functional Plant Biology 41, 963–971. 10.1071/FP14067 32481049

[CIT0071] Schoppach R, Taylor JD, Majerus E, Claverie E, Baumann U, Suchecki R, Fleury D, Sadok W 2016 High resolution mapping of traits related to whole-plant transpiration under increasing evaporative demand in wheat. Journal of Experimental Botany 67, 2847–2860. 27001921 10.1093/jxb/erw125 PMC4861027

[CIT0072] Schymanski SJ, Breitenstein D, Or D 2017 Technical note: an experimental set-up to measure latent and sensible heat fluxes from (artificial) plant leaves. Hydrology and Earth System Sciences 21, 3377–3400.

[CIT0073] Schymanski SJ, Or D 2017 Leaf-scale experiments reveal an important omission in the Penman–Monteith equation. Hydrology and Earth System Sciences 21, 685–706.

[CIT0074] Schymanski SJ, Or D, Zwieniecki M 2013 Stomatal control and leaf thermal and hydraulic capacitances under rapid environmental fluctuations. PLoS One 8, e54231. 23359800 10.1371/journal.pone.0054231 PMC3554716

[CIT0075] Stokes VJ, Morecroft MD, Morison JIL 2006 Boundary layer conductance for contrasting leaf shapes in a deciduous broadleaved forest canopy. Agricultural and Forest Meteorology 139, 40–54.

[CIT0076] Sweet KJ, Peak D, Mott KA 2017 Stomatal heterogeneity in responses to humidity and temperature: testing a mechanistic model. Plant, Cell & Environment 40, 2771–2779. 10.1111/pce.13051 28777880

[CIT0077] Takahashi S, Monda K, Negi J, Konishi F, Ishikawa S, Hashimoto-Sugimoto M, Goto N, Iba K 2015 Natural variation in stomatal responses to environmental changes among *Arabidopsis thaliana* ecotypes. PLoS One 10, e0117449. 25706630 10.1371/journal.pone.0117449 PMC4338149

[CIT0078] Taylor SH, Long SP 2017 Slow induction of photosynthesis on shade to sun transitions in wheat may cost at least 21% of productivity. Philosophical Transactions of the Royal Society B: Biological Sciences 372. doi: 10.1098/rstb.2016.0543. PMC5566890 28808109

[CIT0079] Tilman D, Balzer C, Hill J, Befort BL 2011 Global food demand and the sustainable intensification of agriculture. Proceedings of the National Academy of Sciences, USA 108, 20260–20264. 10.1073/pnas.1116437108 PMC3250154 22106295

[CIT0080] Tilman D, Clark M 2015 Food, agriculture & the environment: can we feed the world & save the earth? Daedalus 144, 8–23.

[CIT0081] Vialet-Chabrand S, Dreyer E, Brendel O 2013 Performance of a new dynamic model for predicting diurnal time courses of stomatal conductance at the leaf level. Plant, Cell & Environment 36, 1529–1546. 10.1111/pce.12086 23448751

[CIT0082] Vialet-Chabrand SRM, Hills A, Wang Y, Griffiths H, Lew V, Lawson T, Blatt MR, Rogers S 2017 *a* Global sensitivity analysis of onguard models identifies key hubs for transport interaction in stomatal dynamics. Plant Physiology 174, 680–688. 28432256 10.1104/pp.17.00170 PMC5462055

[CIT0083] Vialet-Chabrand S, Matthews JSA, Brendel O, Blatt MR, Wang Y, Hills A, Griffiths H, Rogers S, Lawson T 2016 Modelling water use efficiency in a dynamic environment: an example using *Arabidopsis thaliana*. Plant Science 251, 65–74. 27593464 10.1016/j.plantsci.2016.06.016 PMC5038844

[CIT0084] Vialet-Chabrand S, Matthews JS, Simkin AJ, Raines CA, Lawson T 2017 *b* Importance of fluctuations in light on plant photosynthetic acclimation. Plant Physiology 173, 2163–2179. 28184008 10.1104/pp.16.01767 PMC5373038

[CIT0085] Wang Y, Hills A, Blatt MR 2014 Systems analysis of guard cell membrane transport for enhanced stomatal dynamics and water use efficiency. Plant Physiology 164, 1593–1599. 24596330 10.1104/pp.113.233403 PMC3982726

[CIT0086] Wang Y, Holroyd G, Hetherington AM, Ng CK 2004 Seeing ‘cool’ and ‘hot’—infrared thermography as a tool for non-invasive, high-throughput screening of Arabidopsis guard cell signalling mutants. Journal of Experimental Botany 55, 1187–1193. 15073209 10.1093/jxb/erh135

[CIT0087] Way DA, Pearcy RW 2012 Sunflecks in trees and forests: from photosynthetic physiology to global change biology. Tree Physiology 32, 1066–1081. 22887371 10.1093/treephys/tps064

[CIT0088] Weyers JDB, Lawson T 1997 Heterogeneity in stomatal characteristics. Advances in Botanical Research 26, 317–352.

[CIT0089] Weyers JDB, Meidner H 1991 Methods in stomatal research. The Quarterly Review of Biology 66, 492–493.

[CIT0090] Wik M, Pingali P, Broca S 2008 Global agricultural performance: past trends and future prospects. World Development Report 1, 1–39.

[CIT0091] Zeppel MJ, Lewis JD, Phillips NG, Tissue DT 2014 Consequences of nocturnal water loss: a synthesis of regulating factors and implications for capacitance, embolism and use in models. Tree Physiology 34, 1047–1055. 25413023 10.1093/treephys/tpu089

[CIT0092] Zwieniecki MA, Haaning KS, Boyce CK, Jensen KH 2016 Stomatal design principles in synthetic and real leaves. Journal of the Royal Society Interface 13, doi: 10.1098/rsif.2016.0535. PMC5134010 27807270

