# Supplementary material for: Dynamic leaf energy balance: deriving stomatal conductance from thermal imaging in a dynamic environment
Source: J Exp Bot. 2019 Feb 22;70(10):2839–55. doi: 10.1093/jxb/erz068 (PMC6506762; doi:10.1093/jxb/erz068)
Supplement: Supplementary Table S1 and Figures S1-S8 and Protocol_S1 [file erz068_suppl_supplementary_table_s1_figures_s1-s8_protocol_s1.pdf]

Supplementary Table:

Table S1: Equations required to derive environmental variables from the raw environmental measurements collected from various sensors. Temperatures are expressed in °K.

Symbol	Definition	Equation	Units
$e(T)$	Saturation vapor pressure (Buck, 1981)	$e(T) = 0.61365e^{\frac{17.502(T-273.15)}{(T-32.18)}}$	Pa
e_s	Leaf internal vapor pressure	$e_s = e(T_l)$	Pa
e_a	Air vapor pressure	$e_a = e(T_a) \cdot RH$	Pa
$\lambda(T)$	Latent heat of vaporization of water (Henderson-Sellers, 1984)	$\lambda(T) = 1.91846e6 \cdot \left(\frac{T}{T-33.91}\right)^2$	J kg^{-1}
ρ	Air density	$\rho = \frac{P_{atm}}{R_S \cdot T_{air}}$	kg m^{-3}
R_S	Specific gas constant for dry air	$R_S = \frac{R}{M} = 287.058$	J kg^{-1} K^{-1}
M	Molar mass of a gas mixture	$M = 28.9645$ for dry air	g mol^{-1}
SH	Specific humidity	$SH = 0.622 \cdot \frac{e_a}{P_{atm}-e_a}$	kg(H_2O) kg(air)$^{-1}$
C_s	Specific heat capacity of humid air	$C_p + 1820 \cdot SH$	J kg^{-1} K^{-1}

Conductance in the energy balance equations is expressed in m s^{-1} and convert to mol m^{-2} s^{-1} using:

$$g_w(\mathrm{mol\ m^{-2}s^{-1}}) = g_w(\mathrm{m\ s^{-1}}) \cdot \frac{P_{atm}}{RT_{leaf}}$$

where P_{atm} is the atmospheric pressure (Pa), R the gas constant (m^3 Pa K^{-1} mol^{-1}) and T_{leaf} is the leaf temperature (°K).

Supplementary Figures:

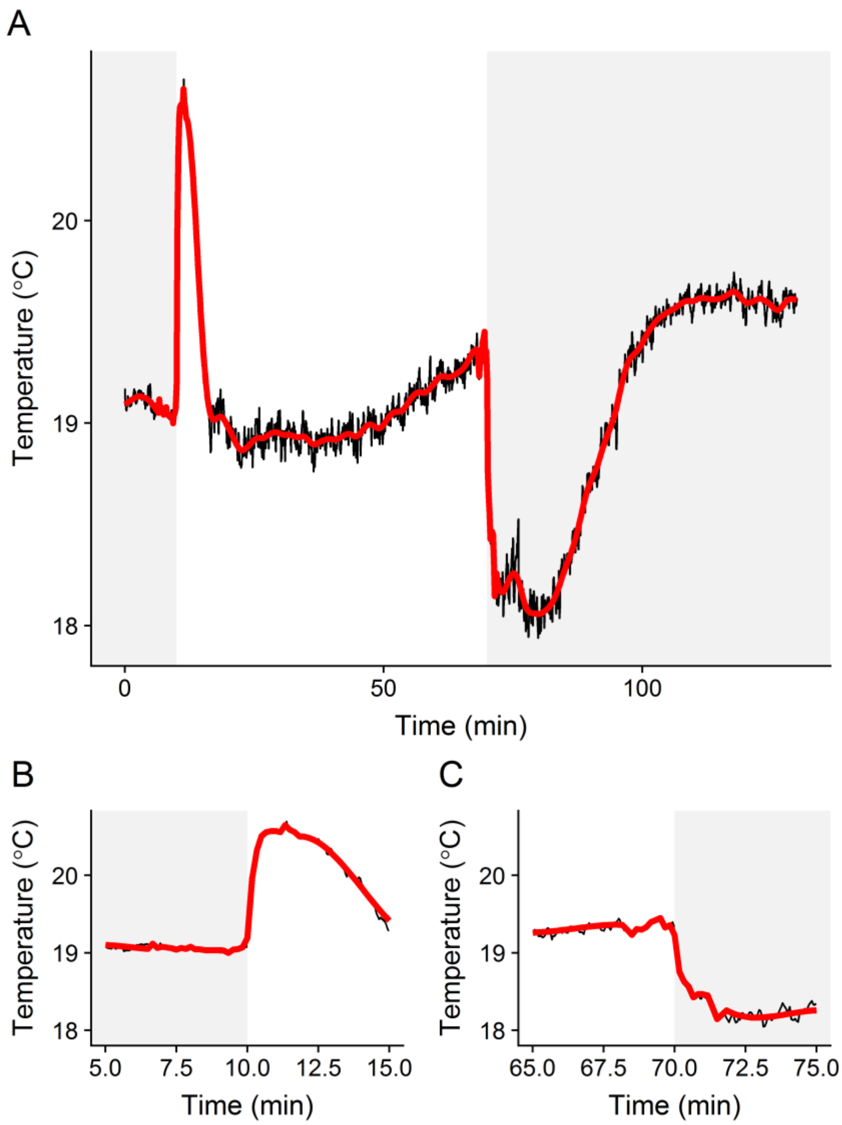

Figure S1: Example of signal processing to remove high frequency noise from infrared thermal measurement. (A) Fitting a cubic smoothing spline (red line) on the observed leaf temperature (black line) removed the high frequency noise and kept the relevant variations. The algorithm used here preserved the rapid increase in temperature happening after a step change in light intensity (B and C). Dark areas represent a period where light intensity was 0 µmol m^{-2} s^{-1} and the white area a period where light intensity was 430 µmol m^{-2} s^{-1}.

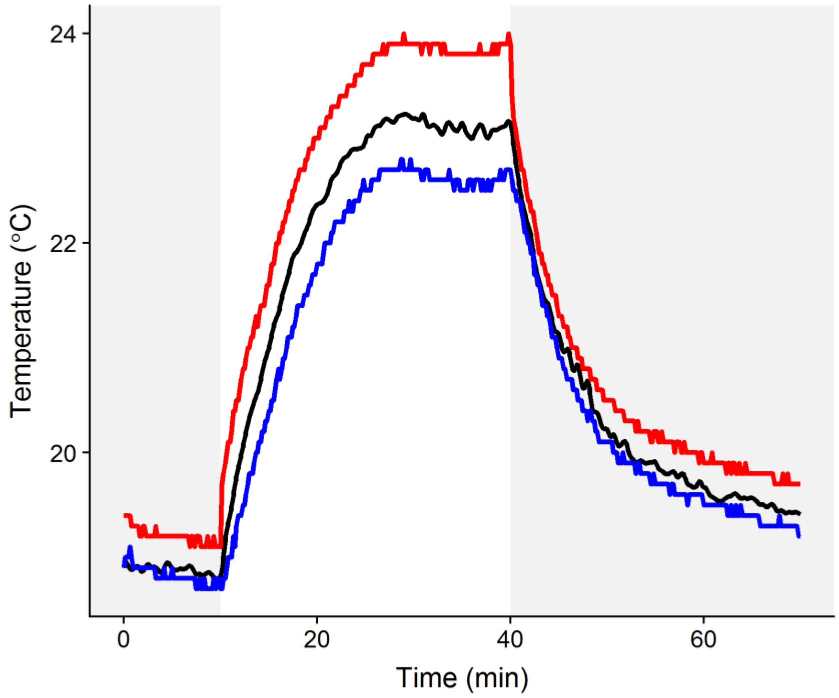

Figure S2: Example of temperature kinetics measured using thermal imaging (black line) or thermocouples (top side: red line, bottom side: blue line) on the reference used to validate accuracy of model predictions. The difference in temperature kinetics was due to the thickness of the replica (c.a. 1.5mm) creating a thermal gradient between both faces: the top face being heated by light energy and the bottom face cooled by transpiration. Thermal imaging captured an average signal between the two faces. Dark areas represent a period where light intensity was 0 µmol m^{-2} s^{-1} and the white area a period where light intensity was 430 µmol m^{-2} s^{-1}.

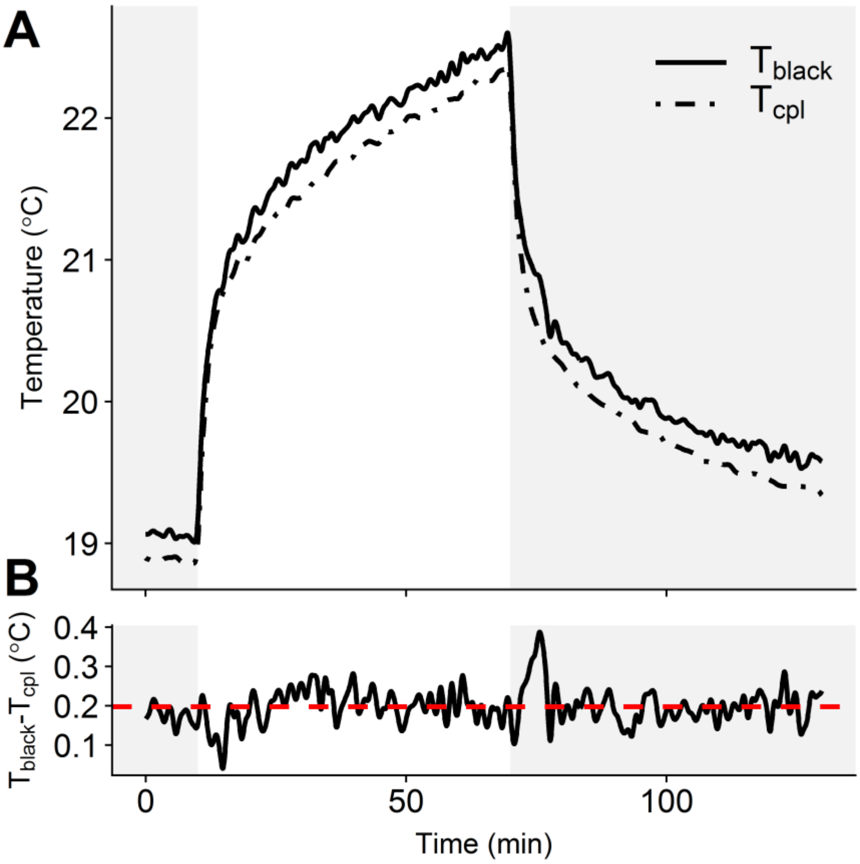

Figure S3: Comparison of temperature measurements using an infrared thermal camera (T_{black}, solid line) and a thermocouple (T_{cpl}, dashed line). (A) A black paint aluminium reference was measured simultaneously on the same area using the two cited methods. (B) The difference in temperature between the two methods were randomly distributed, which signifies that both methods captured the same pattern of variation. The average difference between the two methods (red dashed line) was 0.2°C, which was in the range of the ±0.2°C precision of the methods used here. Dark areas represent a period where light intensity was 0 µmol m^{-2} s^{-1} and the white area a period where light intensity was 430 µmol m^{-2} s^{-1}.

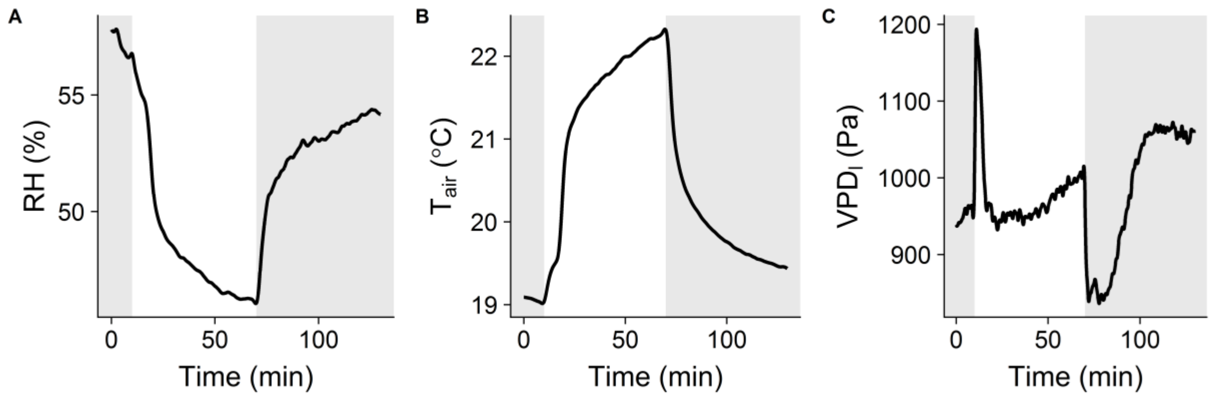

Figure S4: Environmental conditions during step changes of light intensity represented in Fig. 4. (A) Air relative humidity (RH). (B) Air temperature. (C) Example of leaf to air vapour pressure deficit. Dark areas represent a period where light intensity was 0 µmol m^{-2} s^{-1} and the white area a period where light intensity was 430 µmol m^{-2} s^{-1}.

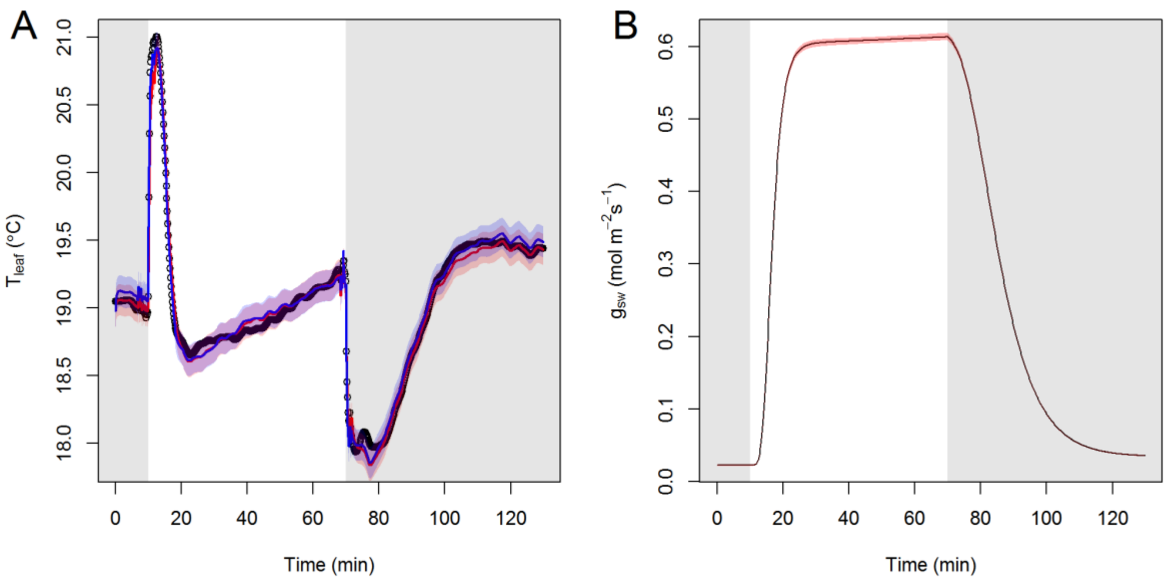

Figure S5: Example of the performance of the energy balance model to reproduce (A) leaf temperature kinetic and (B) stomatal conductance (g_{sw}). (A) The red and blue line represent the leaf temperature predicted using a black and a white reference respectively. The red and blue shaded area represent the 95% confidence interval of the predicted leaf temperature. (B) The solid black line represents the predicted g_{sw} and the red shaded area its 95% confidence interval. Using samples draw from the Bayesian inference, the parameter uncertainty was propagated in g_{sw} calculation and the 95% confidence interval was inferred. Dark areas represent a period where light intensity was 0 µmol m^{-2} s^{-1} and the white area a period where light intensity was 430 µmol m^{-2} s^{-1}.

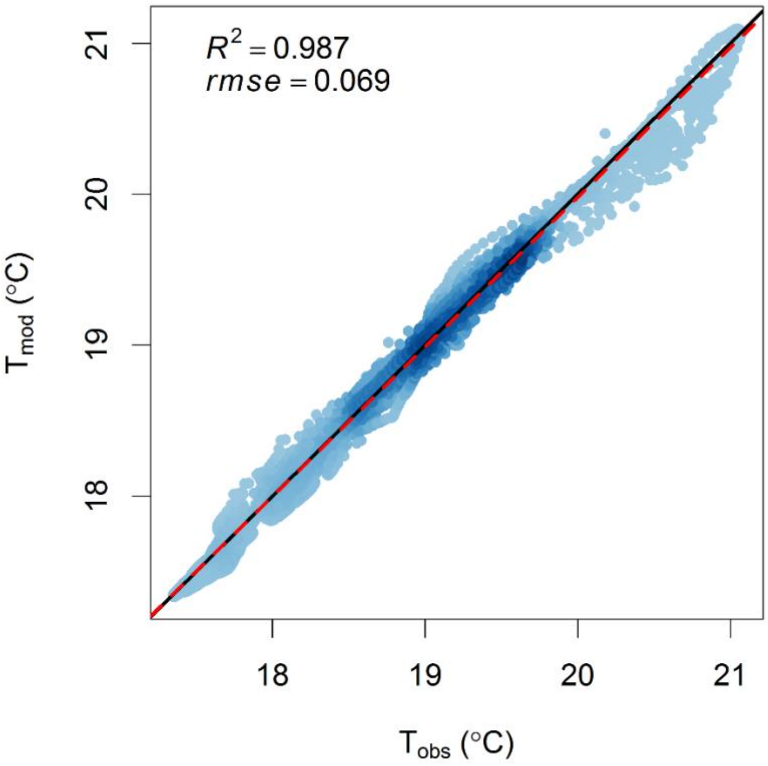

Figure S6: Performance of the energy balance model to reproduce leaf temperature kinetics represented in Fig.4. Observed (T_{obs}) and Modelled (T_{mod}) leaf temperature were compared using a standardized major axis (SMA) regression (red dashed line). The coefficient of determination (R^2) was derived from the regression, as well as the root mean square error (rmse), to characterize the model precision. The 1:1 line (black solid line) was represented to help characterizing the model accuracy.

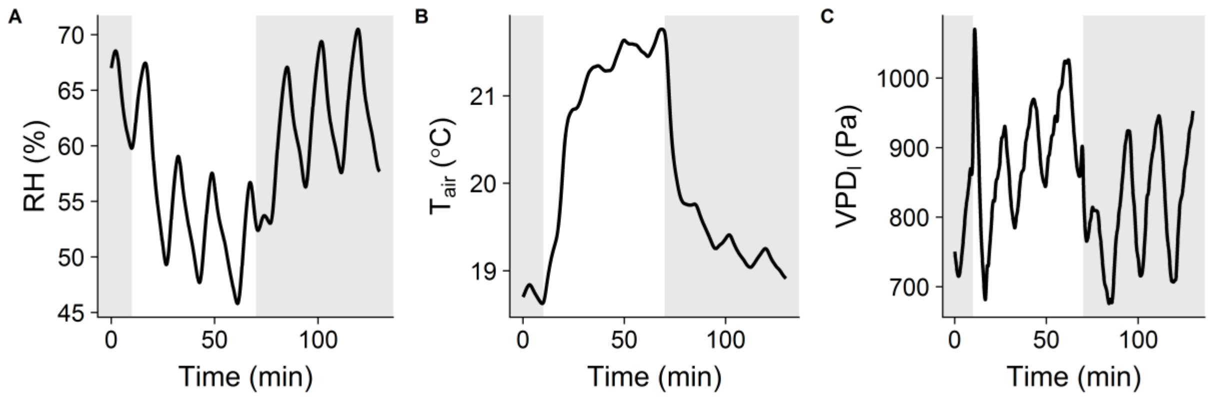

Figure S7: Environmental conditions during step changes of light intensity represented in Fig. 6. (A) Air relative humidity (RH). (B) Air temperature. (C) Example of leaf to air vapour pressure deficit. Dark areas represent a period where light intensity was 0 µmol m^{-2} s^{-1} and the white area a period where light intensity was 430 µmol m^{-2} s^{-1}.

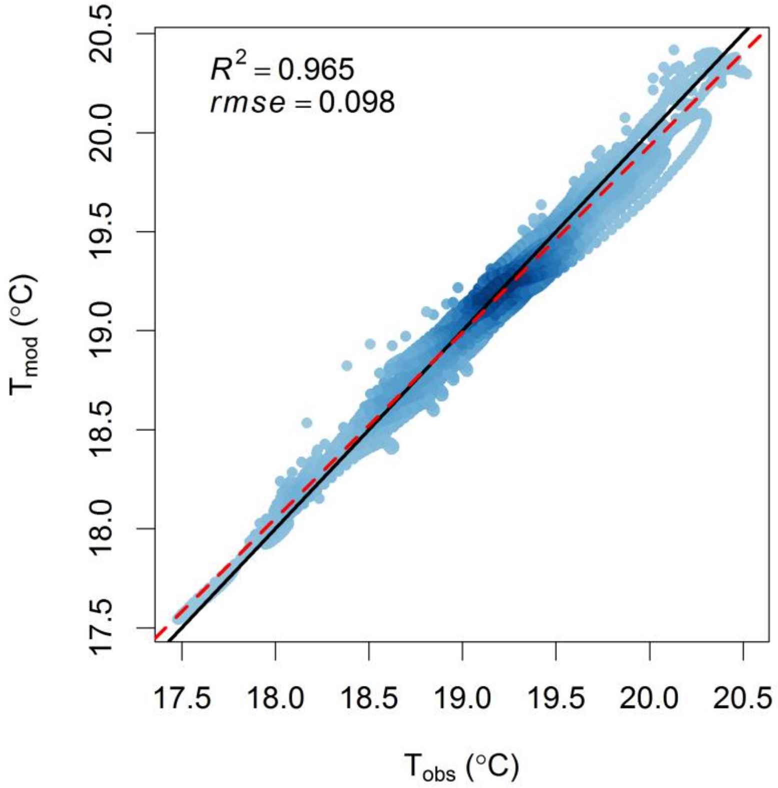

Figure S8: Performance of the energy balance model to reproduce leaf temperature kinetics represented in Fig.6. Observed (T_{obs}) and Modelled (T_{mod}) leaf temperature were compared using a standardized major axis (SMA) regression (red dashed line). The coefficient of determination (R^2) was derived from the regression, as well as the root mean square error (rmse), to characterize the model precision. The 1:1 line (black solid line) was represented to help characterizing the model accuracy.

Algorithm 1: Calculate two estimates of the leaf temperature derivative based on the temperature kinetic of a black and a white reference

<u>function Derivs</u> (t, y, p);
Input : t the current time point in the integration
y the current estimate of the variables in the ODE system
p the parameters for the leaf energy balance model
Output: $\mathrm{d}y/\mathrm{d}t$ the predicted leaf temperature derivatives at time t

1 Assign parameter values proposed by the Bayesian inference algorithm:
$k_{leaf}, g_{bh}^{ref}, \alpha_l, s_l, g_1, g_2, g_3, \lambda_i, k_i, \lambda_d, k_d = p$;

2 Assign current variable values using spline function:
$RH, T_{air}, P_a, PPFD, T_{black}, T_{white} = Spline(t)$;

3 Assign current reference temperature derivative using spline function:
$\mathrm{d}T_{black}/\mathrm{dt}$, $\mathrm{d}T_{white}/\mathrm{dt} = Spline'(\mathrm{t})$;

4 Calculate current stomatal conductance to water vapour:
with: t_1, t_2, t_3 the time at which light intensity was changed
Sc the sigmoidal function describing the temporal response of g_{sw}
if $t < t_1$ **then**
  $g_{sw} = g_1$;
else if $t >= t_1$ *and* $t < t_2$ **then**
  $g_{sw} = Sc(t - t_1, g_1, g_2, \lambda_i, k_i, s_l)$;
else
  $g_{init} = Sc(t_2 - t_1, g_1, g_2, \lambda_i, k_i, s_l)$;
  $g_{sw} = Sc(t - t_2, g_{init}, g_3, \lambda_d, k_d, 0)$;
end

5 Calculate total conductance to water vapour:
$g_{tw} = 1/(1/g_{sw} + 0.92/g_{bh}^{ref})$;

6 Calculate the leaf energy balance variation using a black reference:
$LW_b = 2 \cdot \theta \cdot (\epsilon_b \cdot T_{black}^4 - \epsilon_l \cdot y[1]^4)$;
$SW_b = (\alpha_l - \alpha_b) \cdot I_s$;
$SH_b = 2 \cdot \rho \cdot Cs \cdot g_{bh}^{ref} \cdot (T_{black} - y[1])$;
$LH_b = 2 \cdot \lambda_b \cdot 0.622 \cdot \rho / P_a \cdot g_{tw} \cdot vpd_b$;
$\mathrm{d}y/\mathrm{d}t[1] = (k_{ref} \cdot \mathrm{d}T_{black} + LW_b + SW_b + SH_b - LH_b)/k_{leaf}$;

7 Calculate the leaf energy balance variation using a white reference:
$LW_w = 2 \cdot \theta \cdot (\epsilon_b \cdot T_{white}^4 - \epsilon_l \cdot y[2]^4)$;
$SW_w = (\alpha_l - \alpha_w) \cdot I_s$;
$SH_w = 2 \cdot \rho \cdot Cs \cdot g_{bh}^{ref} \cdot (T_{white} - y[2])$;
$LH_w = 2 \cdot \lambda_w \cdot 0.622 \cdot \rho / P_a \cdot g_{tw} \cdot vpd_w$;
$\mathrm{d}y/\mathrm{d}t[2] = (k_{ref} \cdot \mathrm{d}T_{white} + LW_w + SW_w + SH_w - LH_w)/k_{leaf}$;

return $\mathrm{d}y/\mathrm{d}t$;
